# Assortative mating and differential male mating success in an ash hybrid zone population

**DOI:** 10.1186/1471-2148-6-96

**Published:** 2006-11-15

**Authors:** Pierre R Gérard, Etienne K Klein, Frédéric Austerlitz, Juan F Fernández-Manjarrés, Nathalie Frascaria-Lacoste

**Affiliations:** 1 Laboratoire Ecologie, Systématique, Evolution, UMR ENGREF-CNRS 8079, Bât. 360, Université Paris-Sud, 91405 Orsay Cedex, France; 2 Unité de Biométrie, INRA, Domaine St-Paul, Site Agroparc, 84914 Avignon cedex 9, France

## Abstract

**Background:**

The structure and evolution of hybrid zones depend mainly on the relative importance of dispersal and local adaptation, and on the strength of assortative mating. Here, we study the influence of dispersal, temporal isolation, variability in phenotypic traits and parasite attacks on the male mating success of two parental species and hybrids by real-time pollen flow analysis. We focus on a hybrid zone population between the two closely related ash species *Fraxinus excelsior *L. (common ash) and *F. angustifolia *Vahl (narrow-leaved ash), which is composed of individuals of the two species and several hybrid types. This population is structured by flowering time: the *F. excelsior *individuals flower later than the *F. angustifolia *individuals, and the hybrid types flower in-between. Hybrids are scattered throughout the population, suggesting favorable conditions for their local adaptation. We estimate jointly the best-fitting dispersal kernel, the differences in male fecundity due to variation in phenotypic traits and level of parasite attack, and the strength of assortative mating due to differences in flowering phenology. In addition, we assess the effect of accounting for genotyping error on these estimations.

**Results:**

We detected a very high pollen immigration rate and a fat-tailed dispersal kernel, counter-balanced by slight phenological assortative mating and short-distance pollen dispersal. Early intermediate flowering hybrids, which had the highest male mating success, showed optimal sex allocation and increased selfing rates. We detected asymmetry of gene flow, with early flowering trees participating more as pollen donors than late flowering trees.

**Conclusion:**

This study provides striking evidence that long-distance gene flow alone is not sufficient to counter-act the effects of assortative mating and selfing. Phenological assortative mating and short-distance dispersal can create temporal and spatial structuring that appears to maintain this hybrid population. The asymmetry of gene flow, with higher fertility and increased selfing, can potentially confer a selective advantage to early flowering hybrids in the zone. In the event of climate change, hybridization may provide a means for *F. angustifolia *to further extend its range at the expense of *F. excelsior*.

## Background

Hybrid zones, where lineages differentiable by one or more heritable traits meet and intercross, provide unique opportunities for studying the nature and dynamics of barriers to gene exchange. The evolution of these barriers can have many different outcomes, including divergence of populations leading to speciation, collapse of differentiated populations, hybrid speciation or invasion. The structure and evolution of hybrid zones depend mainly on the relative importance of dispersal, local adaptation and the fitness of hybrids [1,2], influencing the strength of reproductive isolation. For example, with relatively high dispersal between adjacent populations, gene exchange between species is prevented only if local adaptation is sufficiently strong to eliminate hybrids. Temporal isolation is a particular ecological isolation process that can result from divergent adaptations and cause assortative mating by itself. It involves differences in reproductive timing and can be the result of behavioral or developmental schedule divergences [3,4]. In this study we focus mainly on the role of dispersal and temporal assortative mating in shaping the mating patterns in a plant hybrid zone population, and on the relative male fitness of hybrids and parental species.

Pollen dispersal is an important factor influencing the dynamics and evolution of plant populations (e.g. [5,6]). In particular, the frequency of long-distance dispersal events can have strong effects on the distribution of genetic diversity by connecting distant demes in metapopulations [7,8]: in contact zones, it may have important consequences because it can break down the genetic integrity of locally adapted populations and counter-balance the strength of selection. Several processes can limit gene flow despite long-distance dispersal, and thereby increase the efficiency of response to selection. High selfing rates for example may preserve genetic integrity of well-adapted populations, but on the other hand they can also generate inbreeding depression and reduce the effect of selection [9-11]. In plants, temporal assortative mating is usually due to flowering time differences, which can also impede gene flow despite sympatry. If parental species are adapted to different habitats in parapatry, divergence in flowering time can be reinforced in the contact zone, thereby preventing gene flow and maladaptive hybridization. Examples of this can be seen in natural populations of *Agrostis tenuis *and *Anthoxanthum odoratum *at mine boundaries [12] and in *A. odoratum *growing under different treatments in the 150-year old Park Grass Experiment [13]. In contrast, if divergent flowering times are selected in allopatry by different environmental factors, they may overlap in sympatry if intermediate ecotones exist [3] or in the case of habitat disturbance (e.g. [14]). If hybrids do not suffer reduced fitness, the only way to maintain temporal isolation is a variation of selection regimes through the reproductive season [15]. Indeed, as flowering times are often highly heritable, assortative mating due to flowering phenology can strongly influence the response to selection, for example it can increase the rate of response to directional selection [16,17]. Moreover, in the case of strong temporal isolation, high selfing rates can provide reproductive insurance and thus contribute to maintain this isolation.

Local scale studies involving cross-generational approaches with molecular markers are known to be useful for exploring the interactions between selection, assortative mating and dispersal in natural hybrid populations [18]. However, very few recent studies have used methods such as paternity or mating system analyses to estimate the importance of assortment and/or heterogeneity in mating success in structuring hybrid zones of plants (e.g. [19-21]) or animals (e.g. [22]). Detecting temporal assortative mating, i.e. the correlation in flowering time between pollen donors and recipients, can be accomplished at a local scale by paternity analysis [23]. Here we extend a recently developed mating model [24] to estimate the level of temporal assortative mating, along with other important parameters involved in the evolution of a hybrid zone population between two closely related forest tree species.

The two European ash species *Fraxinus excelsior *L. and *F. angustifolia *Vahl have separate distributions in France but occur in sympatry in several contact zones where they hybridize [25], although they show completely disjoint flowering phenologies in allopatry [26,27]. We previously showed that the extent of hybridization seems to be limited by climatic variations in some regions, but intermediate conditions provide ecotones where hybrids are widespread, such as in the Loire valley in central France [25]. Here we focus on one of the populations of this hybrid zone, in which we have shown that genetic and morphological differentiation of the adult trees correlates with differences in flowering times, producing isolation by time patterns [28]. Individuals with extreme phenologies appear genetically and morphologically similar to one parental species, while intermediate flowering individuals cluster into several hybrid classes with flowering dates between those of the two species. Moreover, we showed that these intermediate flowering hybrids produced more flowers and fruits over the two years of study. If these high levels of flowering and fruiting lead to a higher fitness, we may expect that these hybrid genotypes will rapidly invade the zone, especially if assortative mating occurs and/or selfing is frequent. The two parental species are known to be outcrossing species (e.g. *t*_*m *_values provide outcrossing rates between 0.94 and 1 for *F. excelsior *[29,30], and between 0.95 and 1 for *F. angustifolia *[Fernandez-Manjarrés and Gérard, unpublished]) but to date, there have been no study examining the level of selfing in hybrids.

In this paper, we use a modified version of the mixed-mating model [24,31,32] to explore the relative importance of diverse forces influencing the evolution of this hybrid zone population by estimating jointly: (i) the pollen dispersal kernel and the rate of pollen immigration from outside the population, (ii) the strength of spatial and temporal assortative mating, (iii) the selfing rate within the population, (iv) the relative male fitness through mating success of the parental species and different intermediate flowering hybrids and (v) the effect of different phenotypic trait variations on male fecundity (i.e. sexual phenotype, flowering intensity, tree size and fruit production), and (vi) the effect of floral parasite infection intensity on the male fecundity. We also estimate the variation in selfing rates among phenological groups in order to assess the level of selfing in hybrids compared with the parental species. Additionally, we study the impact of the genotyping error rate assumed in the method, as it can have a strong impact on the estimation of relative mating successes [33,34].

## Results

### Joint estimation of the dispersal kernel, temporal assortative mating and male fecundities

#### Dispersal kernel and immigration

**Figure 1:**
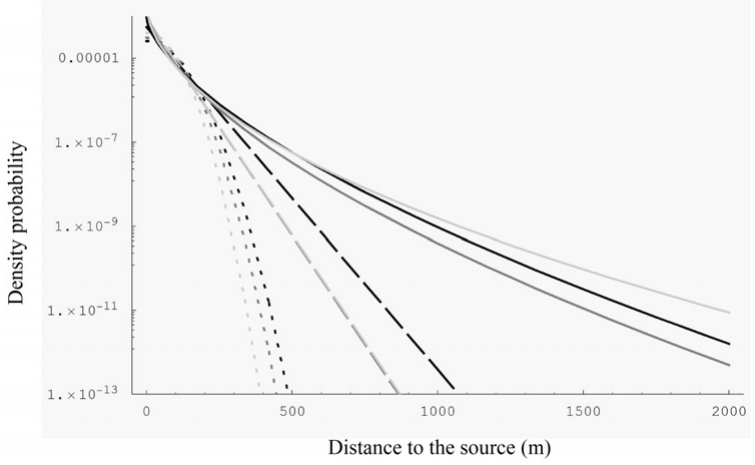
Log-plot of dispersal kernels estimated under the Gaussian (dotted lines), exponential (dashed lines) and exponential power (plain lines) models, without error rate (black) and with low error rate (dark grey) and high error rate (soft grey). All kernels were estimated under the complete model (model 1).

The exponential power dispersal kernel provided here a better fit than the normal or the exponential kernel and than panmixia. Note however that the fit of the exponential power was not significantly better than the exponential kernel assuming a genotyping error rate of 0% and 0.1% (Table 1). The estimated dispersal kernel was fat-tailed: the shape parameters (b^
 MathType@MTEF@5@5@+=feaafiart1ev1aaatCvAUfKttLearuWrP9MDH5MBPbIqV92AaeXatLxBI9gBaebbnrfifHhDYfgasaacH8akY=wiFfYdH8Gipec8Eeeu0xXdbba9frFj0=OqFfea0dXdd9vqai=hGuQ8kuc9pgc9s8qqaq=dirpe0xb9q8qiLsFr0=vr0=vr0dc8meaabaqaciaacaGaaeqabaqabeGadaaakeaacuWGIbGygaqcaaaa@2E09@) were lower than 1 (Table 1). Assuming genotyping error rate of 0.1% or 2.5% decreased the estimates of the scale parameter of the Gaussian and exponential kernels leading to shorter dispersal distances. In contrast, high genotyping error led to fatter-tailed exponential power kernel (i.e. with a smaller shape parameter *b*) and also to a greater mean dispersal distance (Table 1 and Figure 1). The estimated immigration rates decreased when the rate of assumed genotyping errors increased (Table 1), but was still ~60% for the highest assumed error rate.

**Table 1:** Dispersal and selfing parameter estimates

Genotyping error	Dispersal distribution	Parameter estimates					LRT		
				
		-*L*	m^ MathType@MTEF@5@5@+=feaafiart1ev1aaatCvAUfKttLearuWrP9MDH5MBPbIqV92AaeXatLxBI9gBaebbnrfifHhDYfgasaacH8akY=wiFfYdH8Gipec8Eeeu0xXdbba9frFj0=OqFfea0dXdd9vqai=hGuQ8kuc9pgc9s8qqaq=dirpe0xb9q8qiLsFr0=vr0=vr0dc8meaabaqaciaacaGaaeqabaqabeGadaaakeaacuWGTbqBgaqcaaaa@2E1F@ [CI]	s^ MathType@MTEF@5@5@+=feaafiart1ev1aaatCvAUfKttLearuWrP9MDH5MBPbIqV92AaeXatLxBI9gBaebbnrfifHhDYfgasaacH8akY=wiFfYdH8Gipec8Eeeu0xXdbba9frFj0=OqFfea0dXdd9vqai=hGuQ8kuc9pgc9s8qqaq=dirpe0xb9q8qiLsFr0=vr0=vr0dc8meaabaqaciaacaGaaeqabaqabeGadaaakeaacuWGZbWCgaqcaaaa@2E2B@ [CI]	b^ MathType@MTEF@5@5@+=feaafiart1ev1aaatCvAUfKttLearuWrP9MDH5MBPbIqV92AaeXatLxBI9gBaebbnrfifHhDYfgasaacH8akY=wiFfYdH8Gipec8Eeeu0xXdbba9frFj0=OqFfea0dXdd9vqai=hGuQ8kuc9pgc9s8qqaq=dirpe0xb9q8qiLsFr0=vr0=vr0dc8meaabaqaciaacaGaaeqabaqabeGadaaakeaacuWGIbGygaqcaaaa@2E09@ [CI]	δ^ MathType@MTEF@5@5@+=feaafiart1ev1aaatCvAUfKttLearuWrP9MDH5MBPbIqV92AaeXatLxBI9gBaebbnrfifHhDYfgasaacH8akY=wiFfYdH8Gipec8Eeeu0xXdbba9frFj0=OqFfea0dXdd9vqai=hGuQ8kuc9pgc9s8qqaq=dirpe0xb9q8qiLsFr0=vr0=vr0dc8meaabaqaciaacaGaaeqabaqabeGadaaakeaaiiGacuWF0oazgaqcaaaa@2E68@ [CI]	d.f.	*K*	*P*-value
Without	Normal	9590.3	**0.82 **[0.78–0.85]	**0.10 **[0.07–0.13]	-	**98 **[80–135]	1	9.6	< 0.01
	Exponential	9586.1	**0.82 **[0.78–0.85]	**0.10 **[0.07–0.13]	-	**106 **[74–164]	1	1.2	0.29
	Exp. power	9585.5	**0.82 **[0.78–0.85]	**0.10 **[0.07–0.13]	**0.63 **[0.28–1.33]	**147 **[90–1072]	-	-	-
	Spatial panmixia	9612.4	**0.81 **[0.77–0.85]	**0.10 **[0.07–0.13]	-	-	2	53.8	< 10^-10^
									
Low	Normal	9565.5	**0.72 **[0.67–0.76]	**0.11 **[0.09–0.15]	-	**90 **[72–105]	1	25	< 10^-5^
	Exponential	9554.4	**0.72 **[0.67–0.76]	**0.11 **[0.09–0.15]	-	**84 **[66–108]	1	2.8	0.098
	Exp. power	9553.0	**0.72 **[0.67–0.76]	**0.11 **[0.08–0.14]	**0.59 **[0.41–1.02]	**119 **[70–215]	-	-	-
	Spatial panmixia	9615.3	**0.73 **[0.69–0.78]	**0.11 **[0.09–0.15]	-	-	2	124.6	0
									
High	Normal	9207.5	**0.63 **[0.58–0.67]	**0.12 **[0.09–0.15]	-	**78 **[68–98]	1	45.4	< 10^-10^
	Exponential	9188.7	**0.63 **[0.58–0.67]	**0.12 **[0.09–0.15]	-	**84 **[68–104]	1	7.8	< 0.01
	Exp. power	9184.8	**0.62 **[0.57–0.67]	**0.12 **[0.09–0.15]	**0.52 **[0.38–0.86]	**140 **[75–233]	-	-	-
	Spatial panmixia	9291.0	**0.62 **[0.58–0.67]	**0.12 **[0.09–0.15]	-	-	2	212.4	0

Selfing rate (*s*), immigration rate (*m*) and pollen dispersal parameters estimated under models 1 and 2, and confidence intervals at 95% (IC). The quality of fit was evaluated through the log-likelihood (*L*) of the data set under each model, and tested by a Likelihood-Ratio Test (LRT) comparing fits under the nested model and the complete model (Equation 4) with Exp. Power kernel. *K *is the LRT statistics and d.f. the number of degrees of freedom. Values of mean dispersal distance estimates (δ^
 MathType@MTEF@5@5@+=feaafiart1ev1aaatCvAUfKttLearuWrP9MDH5MBPbIqV92AaeXatLxBI9gBaebbnrfifHhDYfgasaacH8akY=wiFfYdH8Gipec8Eeeu0xXdbba9frFj0=OqFfea0dXdd9vqai=hGuQ8kuc9pgc9s8qqaq=dirpe0xb9q8qiLsFr0=vr0=vr0dc8meaabaqaciaacaGaaeqabaqabeGadaaakeaaiiGacuWF0oazgaqcaaaa@2E68@) are expressed in meters

#### Temporal assortative mating

The complete *model 1*, which modelled relative flowering phenology, provided a significantly better fit than the model without any effect of relative phenology when accounting for genotyping error (Table 2). The highest reproductive successes were observed when males flowered slightly earlier than the trees they fertilize (g^
 MathType@MTEF@5@5@+=feaafiart1ev1aaatCvAUfKttLearuWrP9MDH5MBPbIqV92AaeXatLxBI9gBaebbnrfifHhDYfgasaacH8akY=wiFfYdH8Gipec8Eeeu0xXdbba9frFj0=OqFfea0dXdd9vqai=hGuQ8kuc9pgc9s8qqaq=dirpe0xb9q8qiLsFr0=vr0=vr0dc8meaabaqaciaacaGaaeqabaqabeGadaaakeaacuWGNbWzgaqcaaaa@2E13@_-1 _and g^
 MathType@MTEF@5@5@+=feaafiart1ev1aaatCvAUfKttLearuWrP9MDH5MBPbIqV92AaeXatLxBI9gBaebbnrfifHhDYfgasaacH8akY=wiFfYdH8Gipec8Eeeu0xXdbba9frFj0=OqFfea0dXdd9vqai=hGuQ8kuc9pgc9s8qqaq=dirpe0xb9q8qiLsFr0=vr0=vr0dc8meaabaqaciaacaGaaeqabaqabeGadaaakeaacuWGNbWzgaqcaaaa@2E13@_-2_> *g*_0_) or when they belonged to the same phenological group (*g*_0_, fixed here at 1). Relative male fecundities decreased as the pollen-recipient trees flowered earlier than the pollen emitting trees. The fecundity on pollen-recipient trees that differed from 4 phenological groups (i.e. *g*_-4_, the fecundity of *F. angustifolia *males on *F. excelsior *females) was estimated at 0. Accounting for possible genotyping errors did not substantially change the range of values of g^
 MathType@MTEF@5@5@+=feaafiart1ev1aaatCvAUfKttLearuWrP9MDH5MBPbIqV92AaeXatLxBI9gBaebbnrfifHhDYfgasaacH8akY=wiFfYdH8Gipec8Eeeu0xXdbba9frFj0=OqFfea0dXdd9vqai=hGuQ8kuc9pgc9s8qqaq=dirpe0xb9q8qiLsFr0=vr0=vr0dc8meaabaqaciaacaGaaeqabaqabeGadaaakeaacuWGNbWzgaqcaaaa@2E13@, except for g^
 MathType@MTEF@5@5@+=feaafiart1ev1aaatCvAUfKttLearuWrP9MDH5MBPbIqV92AaeXatLxBI9gBaebbnrfifHhDYfgasaacH8akY=wiFfYdH8Gipec8Eeeu0xXdbba9frFj0=OqFfea0dXdd9vqai=hGuQ8kuc9pgc9s8qqaq=dirpe0xb9q8qiLsFr0=vr0=vr0dc8meaabaqaciaacaGaaeqabaqabeGadaaakeaacuWGNbWzgaqcaaaa@2E13@_-1_, which was estimated at a much lower value when no genotyping errors were assumed (Table 2).

**Table 2:** Temporal assortative mating parameter estimates

Genotyping error	Parameter estimates									LRT		
		
	g^ MathType@MTEF@5@5@+=feaafiart1ev1aaatCvAUfKttLearuWrP9MDH5MBPbIqV92AaeXatLxBI9gBaebbnrfifHhDYfgasaacH8akY=wiFfYdH8Gipec8Eeeu0xXdbba9frFj0=OqFfea0dXdd9vqai=hGuQ8kuc9pgc9s8qqaq=dirpe0xb9q8qiLsFr0=vr0=vr0dc8meaabaqaciaacaGaaeqabaqabeGadaaakeaacuWGNbWzgaqcaaaa@2E13@_-4_	g^ MathType@MTEF@5@5@+=feaafiart1ev1aaatCvAUfKttLearuWrP9MDH5MBPbIqV92AaeXatLxBI9gBaebbnrfifHhDYfgasaacH8akY=wiFfYdH8Gipec8Eeeu0xXdbba9frFj0=OqFfea0dXdd9vqai=hGuQ8kuc9pgc9s8qqaq=dirpe0xb9q8qiLsFr0=vr0=vr0dc8meaabaqaciaacaGaaeqabaqabeGadaaakeaacuWGNbWzgaqcaaaa@2E13@_-3 _[CI]	g^ MathType@MTEF@5@5@+=feaafiart1ev1aaatCvAUfKttLearuWrP9MDH5MBPbIqV92AaeXatLxBI9gBaebbnrfifHhDYfgasaacH8akY=wiFfYdH8Gipec8Eeeu0xXdbba9frFj0=OqFfea0dXdd9vqai=hGuQ8kuc9pgc9s8qqaq=dirpe0xb9q8qiLsFr0=vr0=vr0dc8meaabaqaciaacaGaaeqabaqabeGadaaakeaacuWGNbWzgaqcaaaa@2E13@_-2 _[CI]	g^ MathType@MTEF@5@5@+=feaafiart1ev1aaatCvAUfKttLearuWrP9MDH5MBPbIqV92AaeXatLxBI9gBaebbnrfifHhDYfgasaacH8akY=wiFfYdH8Gipec8Eeeu0xXdbba9frFj0=OqFfea0dXdd9vqai=hGuQ8kuc9pgc9s8qqaq=dirpe0xb9q8qiLsFr0=vr0=vr0dc8meaabaqaciaacaGaaeqabaqabeGadaaakeaacuWGNbWzgaqcaaaa@2E13@_-1 _[CI]	*g*_0_	g^ MathType@MTEF@5@5@+=feaafiart1ev1aaatCvAUfKttLearuWrP9MDH5MBPbIqV92AaeXatLxBI9gBaebbnrfifHhDYfgasaacH8akY=wiFfYdH8Gipec8Eeeu0xXdbba9frFj0=OqFfea0dXdd9vqai=hGuQ8kuc9pgc9s8qqaq=dirpe0xb9q8qiLsFr0=vr0=vr0dc8meaabaqaciaacaGaaeqabaqabeGadaaakeaacuWGNbWzgaqcaaaa@2E13@_+1 _[CI]	g^ MathType@MTEF@5@5@+=feaafiart1ev1aaatCvAUfKttLearuWrP9MDH5MBPbIqV92AaeXatLxBI9gBaebbnrfifHhDYfgasaacH8akY=wiFfYdH8Gipec8Eeeu0xXdbba9frFj0=OqFfea0dXdd9vqai=hGuQ8kuc9pgc9s8qqaq=dirpe0xb9q8qiLsFr0=vr0=vr0dc8meaabaqaciaacaGaaeqabaqabeGadaaakeaacuWGNbWzgaqcaaaa@2E13@_+2 _[CI]	g^ MathType@MTEF@5@5@+=feaafiart1ev1aaatCvAUfKttLearuWrP9MDH5MBPbIqV92AaeXatLxBI9gBaebbnrfifHhDYfgasaacH8akY=wiFfYdH8Gipec8Eeeu0xXdbba9frFj0=OqFfea0dXdd9vqai=hGuQ8kuc9pgc9s8qqaq=dirpe0xb9q8qiLsFr0=vr0=vr0dc8meaabaqaciaacaGaaeqabaqabeGadaaakeaacuWGNbWzgaqcaaaa@2E13@_+3_	*g*_+4_	d.f.	*K*	*P*-value
Without	**0**	**0.24 **[0.06–0.96]	**1.15 **[0.05–24.78]	**0.84 **[0.12–5.97]	**1.00**	**0.55 **[0.10–3.19]	**0.59 **[0.03–10.77]	**0**	**-**	5	4.0	0.57
Low	**0**	**0.16 **[0.02–1.30]	**1.44 **[0.80–2.59]	**1.72 **[0.92–3.19]	**1.00**	**0.35 **[0.12–1.01]	**0.15 **[0.02–1.02]	**0**	**-**	5	20.4	< 0.001
High	**0**	**0.17 **[0.08–0.39]	**1.41 **[0.12–16.16]	**1.55 **[0.21–11.25]	**1.00**	**0.51 **[0.21–1.26]	**0.07 **[0.05–0.09]	**0**	**-**	5	37.0	< 10^-5^

Assortative mating parameters relative to phenology (*g*) estimated under complete model (Equation 4) with Exp. Power kernel, and confidence intervals at 95% (CI). Indices for parameter estimates indicate the difference in number of phenological groups between the two parents (Father-Mother). The effect of relative phenology was tested by removing it (i.e. temporal panmixia) and comparing to the complete model by a LRT.

#### Male mating success of phenological groups

**Figure 2:**
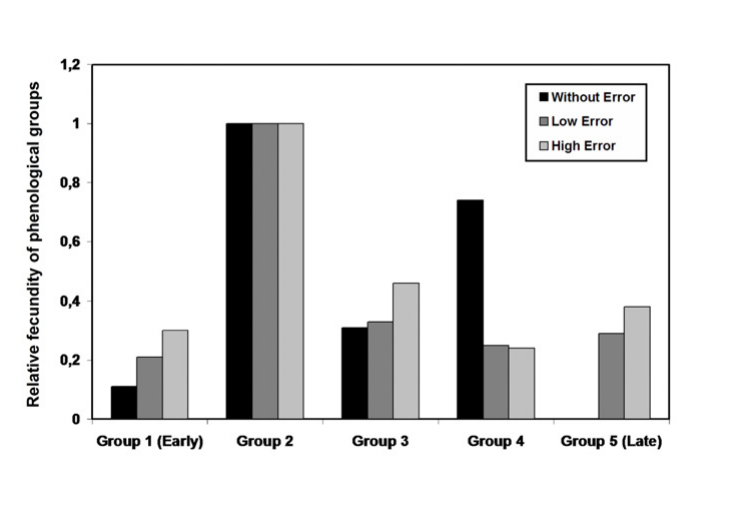
Estimates of relative fecundities of phenological groups, with or without accounting for genotyping error.

The complete *model 2*, where the fertility of each phenological group was estimated over all pollen-recipient trees regardless of their flowering time, provided a significantly better fit than the model without any effect of phenology (*P *< 0.01, *P *< 0.001 and *P *< 0.001 respectively with 0%, 0.1% and 2.5% of genotyping error). The estimated male fecundity was significantly higher for phenological group 2 than for all other groups, and the estimated fecundity of the late flowering "*F. excelsior*" group 5 was zero when assuming no error (Figure 2). All other parameters (dispersal, fecundity, *s *and *m*) did not change compared to *model 1*.

#### Effect of phenotypic trait variations and gall attacks

Flowering intensity and diameter at breast height (DBH) had a significant effect on the relative male fecundities, regardless of the assumed error rate (Table 2): male fecundities were higher for trees with larger DBH and with larger flowering intensities. No other factor had a significant effect, except gall attacks for the assumed error rate of 0.1% (severely attacked trees had lower fecundities), while fruiting intensity had a marginally significant effect (*P*-value = 0.06, higher male fecundity for trees producing more fruits). The effect of the sexual type was never significant. The range of estimated relative fertilities did not change substantially when accounting for genotyping errors, except f^
 MathType@MTEF@5@5@+=feaafiart1ev1aaatCvAUfKttLearuWrP9MDH5MBPbIqV92AaeXatLxBI9gBaebbnrfifHhDYfgasaacH8akY=wiFfYdH8Gipec8Eeeu0xXdbba9frFj0=OqFfea0dXdd9vqai=hGuQ8kuc9pgc9s8qqaq=dirpe0xb9q8qiLsFr0=vr0=vr0dc8meaabaqaciaacaGaaeqabaqabeGadaaakeaacuWGMbGzgaqcaaaa@2E11@_4 _for flowering intensity, f^
 MathType@MTEF@5@5@+=feaafiart1ev1aaatCvAUfKttLearuWrP9MDH5MBPbIqV92AaeXatLxBI9gBaebbnrfifHhDYfgasaacH8akY=wiFfYdH8Gipec8Eeeu0xXdbba9frFj0=OqFfea0dXdd9vqai=hGuQ8kuc9pgc9s8qqaq=dirpe0xb9q8qiLsFr0=vr0=vr0dc8meaabaqaciaacaGaaeqabaqabeGadaaakeaacuWGMbGzgaqcaaaa@2E11@_3 _for gall attacks with a high error and all fertilities for sexual type (particularly f^
 MathType@MTEF@5@5@+=feaafiart1ev1aaatCvAUfKttLearuWrP9MDH5MBPbIqV92AaeXatLxBI9gBaebbnrfifHhDYfgasaacH8akY=wiFfYdH8Gipec8Eeeu0xXdbba9frFj0=OqFfea0dXdd9vqai=hGuQ8kuc9pgc9s8qqaq=dirpe0xb9q8qiLsFr0=vr0=vr0dc8meaabaqaciaacaGaaeqabaqabeGadaaakeaacuWGMbGzgaqcaaaa@2E11@_1 _corresponding to the relative fertility of males) (Table 3).

**Table 3:** Fertility parameter estimates

Genotyping error		Parameter estimates					LRT		
			
	Phenotypical trait	f^ MathType@MTEF@5@5@+=feaafiart1ev1aaatCvAUfKttLearuWrP9MDH5MBPbIqV92AaeXatLxBI9gBaebbnrfifHhDYfgasaacH8akY=wiFfYdH8Gipec8Eeeu0xXdbba9frFj0=OqFfea0dXdd9vqai=hGuQ8kuc9pgc9s8qqaq=dirpe0xb9q8qiLsFr0=vr0=vr0dc8meaabaqaciaacaGaaeqabaqabeGadaaakeaacuWGMbGzgaqcaaaa@2E11@_1 _[CI]	f^ MathType@MTEF@5@5@+=feaafiart1ev1aaatCvAUfKttLearuWrP9MDH5MBPbIqV92AaeXatLxBI9gBaebbnrfifHhDYfgasaacH8akY=wiFfYdH8Gipec8Eeeu0xXdbba9frFj0=OqFfea0dXdd9vqai=hGuQ8kuc9pgc9s8qqaq=dirpe0xb9q8qiLsFr0=vr0=vr0dc8meaabaqaciaacaGaaeqabaqabeGadaaakeaacuWGMbGzgaqcaaaa@2E11@_2 _[CI]	f^ MathType@MTEF@5@5@+=feaafiart1ev1aaatCvAUfKttLearuWrP9MDH5MBPbIqV92AaeXatLxBI9gBaebbnrfifHhDYfgasaacH8akY=wiFfYdH8Gipec8Eeeu0xXdbba9frFj0=OqFfea0dXdd9vqai=hGuQ8kuc9pgc9s8qqaq=dirpe0xb9q8qiLsFr0=vr0=vr0dc8meaabaqaciaacaGaaeqabaqabeGadaaakeaacuWGMbGzgaqcaaaa@2E11@_3 _[CI]	f^ MathType@MTEF@5@5@+=feaafiart1ev1aaatCvAUfKttLearuWrP9MDH5MBPbIqV92AaeXatLxBI9gBaebbnrfifHhDYfgasaacH8akY=wiFfYdH8Gipec8Eeeu0xXdbba9frFj0=OqFfea0dXdd9vqai=hGuQ8kuc9pgc9s8qqaq=dirpe0xb9q8qiLsFr0=vr0=vr0dc8meaabaqaciaacaGaaeqabaqabeGadaaakeaacuWGMbGzgaqcaaaa@2E11@_4 _[CI]	f^ MathType@MTEF@5@5@+=feaafiart1ev1aaatCvAUfKttLearuWrP9MDH5MBPbIqV92AaeXatLxBI9gBaebbnrfifHhDYfgasaacH8akY=wiFfYdH8Gipec8Eeeu0xXdbba9frFj0=OqFfea0dXdd9vqai=hGuQ8kuc9pgc9s8qqaq=dirpe0xb9q8qiLsFr0=vr0=vr0dc8meaabaqaciaacaGaaeqabaqabeGadaaakeaacuWGMbGzgaqcaaaa@2E11@_5 _[CI]	d.f.	*K*	*P*-value
Without	Flowering intensity	**0.06 **[0.01–1.11]	**0.34 **[0.04–3.26]	**0.91 **[0.10–8.02]	**0.63 **[0.06–6.23]	**1.00**	4	12.6	< 0.05
	DBH	**0**	**0.21 **[0.04–1.02]	**0.68 **[0.20–2.30]	**1.31 **[0.44–3.90]	**1.00**	3	24.2	< 10^-3^
	Fruiting intensity	**0.47 **[0.03–8.27]	**0.57 **[0.05–6.19]	**0.30 **[0.03–3.02]	**0.35 **[0.03–3.47]	**1.00**	4	2.6	0.64
	Gall attacks	**1.00**	**1.56 **[0.32–7.65]	**1.71 **[0.52–5.61]	**0.41 **[0.07–2.21]	**0**	3	5.0	0.18
	Sexual type	**0**	**1.00**	**0.97 **[0.13–7.32]	**1.14 **[0.16–8.33]	-	2	1.4	0.51
									
Low	Flowering intensity	**0.27 **[0.07–1.02]	**0.53 **[0.19–1.48]	**0.92 **[0.35–2.43]	**1.72 **[0.66–4.51]	**1.00**	4	16.0	< 0.01
	DBH	**0**	**0.28 **[0.10–0.78]	**0.62 **[0.24–1.58]	**1.39 **[0.61–3.14]	**1.00**	3	34.4	< 10^-5^
	Fruiting intensity	**0.10 **[0.02–0.39]	**0.31 **[0.12–0.85]	**0.16 **[0.05–0.48]	**0.22 **[0.08–0.58]	**1.00**	4	9.2	0.06
	Gall attacks	**1.00**	**2.21 **[0.80–6.15]	**2.48 **[1.13–5.45]	**0.90 **[0.29–2.76]	**0**	3	8.2	< 0.05
	Sexual type	**12.01 **[1.12–128.30]	**1.00**	**1.14 **[0.31–4.20]	**0.84 **[0.24–2.97]	-	3	2.2	0.54
									
High	Flowering intensity	**0.18 **[0.04–0.79]	**0.35 **[0.10–1.27]	**0.84 **[0.26–2.78]	**1.67 **[0.50–5.63]	**1.00**	4	35.2	< 10^-5^
	DBH	**0.03 **[0.01–0.28]	**0.34 **[0.15–0.79]	**0.84 **[0.41–1.75]	**1.24 **[0.62–2.50]	**1.00**	4	44.0	< 10^-5^
	Fruiting intensity	**0.28 **[0.05–1.54]	**0.71 **[0.20–2.47]	**0.37 **[0.11–1.26]	**0.49 **[0.15–1.62]	**1.00**	4	7.8	0.10
	Gall attacks	**1.00**	**2.16 **[0.76–6.18]	**1.71 **[0.85–3.47]	**1.50 **[0.71–3.17]	**0.60 **[0.07–5.22]	4	4.0	0.43
	Sexual type	**12.38 **[0.82–186.18]	**1.00**	**1.61 **[0.21–12.64]	**1.34 **[0.17–10.39]	-	3	5.4	0.15

Fertility parameters relative to phenotypic traits and gall attacks (*f*) estimated under complete model (Equation 4) with Exp. Power kernel. The effect of each factor was tested by removing the factor and comparing to the complete model with all factors by a LRT. Indices indicate different levels of each factor (Flowering and Fruiting intensities: 1 = Anecdotal, 2 = Low, 3 = Intermediate, 4 = Abundant, 5 = Massive; DBH: 1 = < 40 cm, 2 = < 80 cm, 3 = < 120 cm, 4 = < 160 cm, 5 = > 200 cm; Gall attacks: 1 = Inexistent, 2 = Low, 3 = Intermediate, 4 = High, 5 = Massive; Sexual type: 1 = Pure males (MM), 2 = Hermaphrodites with a high proportion of male flowers (MH), 3 = Hermaphrodites with a low proportion of male flowers (HM), 4 = Pure hermaphrodites (HH).

### Male effective population density

With *model 1*, we estimated from equation (8) the reduction of effective male population density (d^
 MathType@MTEF@5@5@+=feaafiart1ev1aaatCvAUfKttLearuWrP9MDH5MBPbIqV92AaeXatLxBI9gBaebbnrfifHhDYfgasaacH8akY=wiFfYdH8Gipec8Eeeu0xXdbba9frFj0=OqFfea0dXdd9vqai=hGuQ8kuc9pgc9s8qqaq=dirpe0xb9q8qiLsFr0=vr0=vr0dc8meaabaqaciaacaGaaeqabaqabeGadaaakeaacuWGKbazgaqcaaaa@2E0D@_*em*_/*d*_*obs*_) when only accounting for variation in phenotypic traits as 0.32 when the assumed genotyping error rate was set at 0%, 0.24 for a rate of 0.1%, and 0.27 for a rate of 2.5%.

The mean reduction of effective male population density due to phenotypic traits and temporal assortative mating was estimated at (d^
 MathType@MTEF@5@5@+=feaafiart1ev1aaatCvAUfKttLearuWrP9MDH5MBPbIqV92AaeXatLxBI9gBaebbnrfifHhDYfgasaacH8akY=wiFfYdH8Gipec8Eeeu0xXdbba9frFj0=OqFfea0dXdd9vqai=hGuQ8kuc9pgc9s8qqaq=dirpe0xb9q8qiLsFr0=vr0=vr0dc8meaabaqaciaacaGaaeqabaqabeGadaaakeaacuWGKbazgaqcaaaa@2E0D@_*em*_/*d*_*obs*_) = 0.30, 0.23 and 0.27 (with an error rate of 0%, 0.1% and 2.5% respectively). Among pollen-recipients, regardless of genotyping error, the highest reductions were estimated from the pollen clouds of pollen-recipients belonging to the two latest phenological groups (4 and 5), and particularly that of group 5 (almost two-fold decrease, results not shown).

Finally, the mean reduction of effective male population density due to phenotypic traits, temporal assortative mating and non-random dispersal was estimated to (d^
 MathType@MTEF@5@5@+=feaafiart1ev1aaatCvAUfKttLearuWrP9MDH5MBPbIqV92AaeXatLxBI9gBaebbnrfifHhDYfgasaacH8akY=wiFfYdH8Gipec8Eeeu0xXdbba9frFj0=OqFfea0dXdd9vqai=hGuQ8kuc9pgc9s8qqaq=dirpe0xb9q8qiLsFr0=vr0=vr0dc8meaabaqaciaacaGaaeqabaqabeGadaaakeaacuWGKbazgaqcaaaa@2E0D@_*em*_/*d*_*obs*_) = 0.09, 0.07 and 0.06 with an error rate of 0%, 0.1% and 2.5% respectively.

### Selfing rates

**Figure 3:**
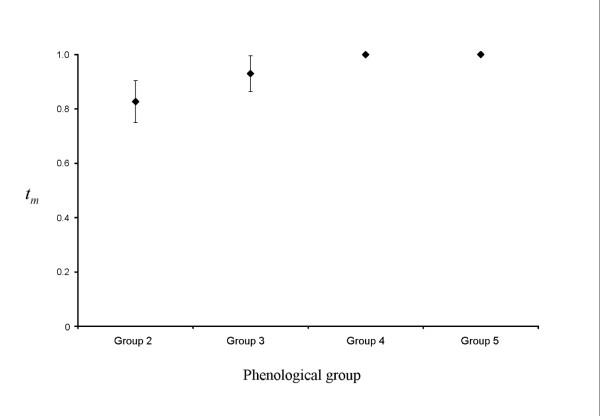
Outcrossing rates *t*_*m *_estimated from family arrays sampled on mothers from each phenological group (2 to 5). Standard errors were computed from 1000 bootstrap replicates over families.

The overall selfing rate estimated with *model 1*or *2 *was 10% and was slightly affected by genotyping error (Table 1). It varied among phenological groups (Figure 3) and among families, as estimated by MLTR: the mean outcrossing rate (*t*_*m*_) was significantly different from 1 within half of the families, with values ranging from 31% to 88% (i.e. selfing rates ranging from 12% to 69%). The selfing rate was close to 20% in group 2 (*t*_*m *_= 82.7%, standard deviation = 7.7%). In group 3, the selfing rate was estimated at 7% (*t*_*m *_= 93%, SD = 0.066): the family-level *t*_*m *_values were significantly different from 1 within 3/8 families, ranging between 62.5% and 94%. The selfing rate was estimated at zero in the two latest flowering groups (4 and 5).

## Discussion

### Methodological insights

According to Araki and Blouin [33], assignment error can have a strong effect on the estimation of the relative reproductive successes of different groups, particularly by increasing type II errors (assignment of an untrue parent) when the proportion of unsampled parents is high. Avoiding these errors is generally difficult [35]. Here, we accounted for genotyping errors more realistically than previous methods [36], where a mistyped allele can be of any size compared to the original allele. Instead, we assumed that a genotyping error could be only of ± 1 repeat unit, which is more realistic for microsatellite loci.

As expected, some estimated parameter values varied with different assumed rates of error. However, the general trends remained quite similar: the estimates of dispersal parameters were relatively stable, and the ranking of relative phenology parameters, as well as fertility parameters of various phenotypic trait classes, were barely affected, except for sexual type. The main difference lay in the significance of effects, which was certainly due to the increased information provided by accounting for genotyping errors.

Similarly, the value of the estimated rate of external pollen (*m*) when genotyping errors were assumed may be more reliable than without errors. The very high value (~80%) obtained when these errors were ignored illustrates a particular caveat of paternity analyses using microsatellite markers: higher allele numbers generally produce higher estimated immigration rates. Indeed, using paternity exclusion methods, the apparent gene flow was estimated here between 45% and 55% with four loci, depending on the level of polymorphism of the selected loci, and increased up to 70% with six loci (Gérard et al., unpublished results). Increasing the level of polymorphism of the chosen loci improves the stringency of paternity analysis, by reducing the rate of multiple assignments and type II errors. At the same time, however, more polymorphic loci increase the risk of overestimating external pollen flow by increasing the error rate.

### Homogenizing effect of long-distance pollen dispersal

We found a high level of pollen immigration, even when typing errors were accounted for (external pollen flow ~60% for the highest assumed genotyping error rate). This has also been found in other anemophilous forest tree species such as *Pseudotsuga menziesii *[37], or *Quercus robur *and *Q. petraea *[38]. Moreover, the best-fitting dispersal kernels were rather fat-tailed, which appears increasingly as a common feature in plants, including perennial herbs (e.g. [39,40]), crops (e.g. [41]) and forest trees (e.g. [42,43]). Long-distance dispersal may tend to connect distant populations, homogenizing differentiated gene pools. Here it is likely to have acted on the evolution of the hybrid zone of the two *Fraxinus *species, by connecting remote populations, increasing local genetic diversity [7] and possibly counteracting the effect of local adaptation if assortative mating is not strong enough. This long-distance dispersal has probably contributed to the creation and maintenance of a large-scale hybrid zone, as detected all along the Loire valley [25].

### Isolating role of assortative mating and selfing

#### Spatial assortative mating

Contrary to long-distance dispersal, short-distance dispersal may limit gene exchange at a local scale. The estimate of the shape parameter *b *of the exponential power kernel was not much smaller than 1 (the value for an exponential kernel) and the estimated mean dispersal distance was quite low (100 <δ^
 MathType@MTEF@5@5@+=feaafiart1ev1aaatCvAUfKttLearuWrP9MDH5MBPbIqV92AaeXatLxBI9gBaebbnrfifHhDYfgasaacH8akY=wiFfYdH8Gipec8Eeeu0xXdbba9frFj0=OqFfea0dXdd9vqai=hGuQ8kuc9pgc9s8qqaq=dirpe0xb9q8qiLsFr0=vr0=vr0dc8meaabaqaciaacaGaaeqabaqabeGadaaakeaaiiGacuWF0oazgaqcaaaa@2E68@ < 150 m, depending of the typing error rate). These findings are consistent with a previous study we conducted in the same population showing that co-flowering trees were patchily distributed in space [28]. Thus, even if long-distance dispersal takes place at a non-negligible rate, a substantial number of reproductive events inside the stand may occur within the patches. Short-distance may produce pronounced spatial genetic structure, as previously detected in this population [28].

#### Temporal assortative mating

The homogenizing effect of long-distance dispersal may also be counteracted by temporal assortative mating. Even with incomplete assortative mating, a large part of inside-stand reproductive events occurred within the same phenological group, which is strengthened by the high level of selfing. These patterns may contribute to maintain isolation by time patterns [28] and potentially increase the rate of response to selection [16,17].

One of our main results is that we detected no crosses between the parental species, as expected considering their phenologies. Thus, intermediate flowering hybrids could represent bridges to gene flow in the contact zone, as described for example for *Asclepias *species [44]. Incidentally, the absence of mating events observed here between the two distinct species raises the question of how the first hybrids were produced. This could be due to exceptional events of hybridization between the two species, which occur at too low a rate to be detected in our study. For example, exceptionally hard winters, in which *F. angustifolia *individuals may have flowered much later than usual, as they require particular heat and chill conditions to flower [26], could have favoured the first hybridization events.

#### Selfing rates

High selfing rates may also contribute to counteract the homogenizing effect of long-distance dispersal, and thus help maintain temporal isolation and increase the success of well adapted genotypes if they do not suffer strong inbreeding depression. Indeed, it has been shown that a mutant exhibiting high selfing rates can invade locally stable outcrossing populations despite strong inbreeding depression [45]. The relatively high rates of self-fertilization of hybrids may provide an advantage for them in colonizing the hybrid zone, as the parental species show very limited selfing: the estimated rates vary between 0 and 6% for *F. excelsior *[29,30], and between 0 and 5% for *F. angustifolia *(Fernandez-Manjarrés and Gérard, unpublished). Given the low selfing rates of parental species, the occurrence of relatively high rates within intermediate flowering groups is surprising. The hypothesis of pollen limitation can be excluded for three reasons: (i) the number of individuals and the mean flowering intensities were higher in the groups where high selfing rates were detected [28], (ii) the reduction in effective male population density due to fertility parameters and phenology was smaller in pollen clouds of mothers from group 2 and 3 (d^
 MathType@MTEF@5@5@+=feaafiart1ev1aaatCvAUfKttLearuWrP9MDH5MBPbIqV92AaeXatLxBI9gBaebbnrfifHhDYfgasaacH8akY=wiFfYdH8Gipec8Eeeu0xXdbba9frFj0=OqFfea0dXdd9vqai=hGuQ8kuc9pgc9s8qqaq=dirpe0xb9q8qiLsFr0=vr0=vr0dc8meaabaqaciaacaGaaeqabaqabeGadaaakeaacuWGKbazgaqcaaaa@2E0D@_*em*_/*d*_*obs*_) = 0.31 and 0.33 respectively), and (iii) mothers showing the highest selfing rates were not those that had the strongest reduction in their number of effective sires (results not shown). A possible explanation for this increased selfing may be a breakdown of epistasis in intermediate flowering hybrids, caused by linkage disequilibrium between alleles at loci involved in self-rejection mechanisms that co-evolved independently within the two species.

### Male mating success of hybrids and parental species

The fertility of hybrids (i.e. the intermediate phenological groups), and in particular individuals from group 2, is higher than the fertility of either parental species (Groups 1 and 5). Differences in some phenotypic traits may participate in this increased fertility.

We found in our previous study [28] that intermediate flowering hybrids (groups 2–4) produced more flowers and had a large DBH (Table 4). Here we show that larger tree diameters and higher flowering intensities increased male fertility (Table 3), as expected for wind-pollinated species [46], and also detected in other tree species (e.g. [24]). This may contribute to increase the fertility of group 2.

**Table 4:** Summary of phenotypic trait values in the phenological groups

	Group 1 (Early)	Group 2	Group 3	Group 4	Group 5 (Late)
Number of individuals	34	95	62	46	32

DBH	111.85 (9.50)	92.44 (4.71)	77.03 (6.44)	78.80 (7.86)	54.53 (8.38)
Flowering intensity	1.50 (0.13)	2.65 (0.11)	2.69 (0.12)	2.52 (0.16)	1.62 (0.19)
Fruiting intensity	0.65 (0.11)	1.54 (0.10)	1.87 (0.13)	1.83 (0.17)	0.94 (0.21)
Gall attacks	0.35 (0.14)	0.27 (0.08)	0.49 (0.13)	0.67 (0.17)	0.53 (0.21)
Morphology (CDA 1)	-0.67 (0.13)	-0.48 (0.10)	0.12 (0.14)	0.26 (0.17)	0.93 (0.14)

Mean values (standard errors) of different phenotypic traits in the five phenological groups. The data were taken from Gérard et al. [28]. Morphology values are the mean coordinates for the first canonical variable from a Canonical Discriminant Analysis. This analysis was performed on four morphological variables in populations of the two species and phenological groups (see [28] for further details).

Surprisingly, we did not detect any effect of the sexual type on fertility, probably due to the similar values of siring success of different classes of hermaphrodites (i.e. individuals with a majority of staminate flowers did not sire more seeds than perfect hermaphrodites). Thus male mating success did not depend on the relative proportions of staminate vs. perfect flowers. Nevertheless, a higher male fertility of males relative to hermaphrodites was detected in controlled crosses [47] and natural populations [30] of *F. excelsior*. This was retrieved here by the very high relative male fecundity of pure males estimated with a typing error rate but it should be nuanced by the very low frequency of males in the population (2%).

Optimal sex allocation may also contribute to increase the male fertility of hybrids. Indeed, trees producing many seeds also had the highest relative male fecundity. Our results are consistent with classical predictions of sex allocation theory, i.e. a constant optimal sex allocation for simultaneous hermaphroditism, where individuals simultaneously produce male and female gametes [48,49]. Nevertheless, many plant species seem to exhibit a gradual shift in sex allocation, and thereby in functional gender, with increasing size [46,50,51]. A positive correlation between male and female reproductive success has been detected in very few cases only [52-54]. Here we present a case where individuals with a high male mating success also show a high female success. Further long-term work is required to confirm that this observation remains true through time and for other hybrid populations.

The higher relative fertility of group 2 may be partly influenced by its lower mean level of gall attacks, compared with those of the other groups [28]. As gall mites (*Eriophyes fraxiniflora*) infect male ash flowers [55], it is expected that high attack levels would influence the relative male fecundity. Indeed, low levels of gall attacks seemed to have little effect but high rates strongly decreased relative fecundity.

### Asymmetry of pollen flow and evolution of the hybrid zone

Flowering phenology also generates asymmetrical pollen flow: trees are quite successful in fertilizing the individuals with a flowering period that overlaps their own but starts later. In contrast, they show quite limited success on the individuals that flower earlier than they do, even if the flowering times overlap. This may result from the protogyny of the *Fraxinus *species: early flowering trees may participate in reproduction mainly as pollen donors. Mite attacks may also contribute to the observed asymmetry of gene flow by pollen since late flowering trees are more infected than early flowering trees [28]. They may also favour hybridization, by reducing the male fertility of late flowering trees. This asymmetry may provide a demographic advantage to early flowering hybrids because of the greater distances of pollen dispersal compared to seed dispersal in forest trees. On the other hand, although *F. angustifolia *individuals (Group1) had poor fertility during the year of study (which is likely due to higher susceptibility to late winter frosts, also affecting their fruit production), they may benefit from favourable years and contribute significantly to young seedling generations (Gérard et al. in preparation). Moreover, *F. angustifolia *shows reduced dormancy compared to *F. excelsior *[56], possibly conferring another demographic advantage. Thus, the invasive potential of *F. angustifolia *through hybridization in this region may be highly modified in the case of global warming.

Further work, however, is still needed to assess the relative fitness of seeds produced by different types of individuals, either selfed or outcrossed. Moreover, as pollen dispersal seems to occur over large distances, a larger sampling effort is perhaps needed to get more accurate estimates of the dynamics of the metapopulation in this hybrid zone. As the evolution of partially cross-fertile plant communities is greatly influenced by the strength of assortative mating and demographic characteristics [57], theoretical work is also needed to better understand the interaction of short- and long-distance dispersal and assortative mating, as well as environmental fluctuations (e.g. climate) in plant hybrid zones.

## Conclusion

Temporal and spatial assortative mating limit gene flow in this hybrid zone population, even if long-distance dispersal should tend to counter-act their effects. Gene flow between parental species does not occur and intermediate flowering hybrids apparently represent bridges to gene flow between them. Early flowering hybrids, which have the highest male mating success, show optimal sex allocation which, with increasing selfing rates, can potentially confer to them a selective advantage in the hybrid zone. Moreover, temporal assortative mating could contribute to increasing the rate of response to selection by limiting gene flow between different classes of individuals.

The asymmetry of gene flow coming from early flowering pollen donors into late flowering recipients is probably a key factor involved in the dynamics and evolution of this hybrid population. If climate warming allows *F. angustifolia *not to suffer from winter frosts, the presence of hybrids could contribute to extending its range through this asymmetry. This study has strong implications for understanding the dynamics of forest hybrid zones and for the management of forest diversity in a climate change context.

## Methods

### Focus species and sampling

Common ash (*F. excelsior *L.) and narrow-leaved ash (*F. angustifolia *Vahl) are closely related species [58] with contrasted distributions across Europe: *F. angustifolia *has a Southern Mediterranean distribution, whereas *F. excelsior *occurs at more Northerly latitudes. They are in sympatry in several regions in France, such as the Loire and Saône valleys [25,59]. Both species are post-pioneer forest trees with a colonizing behavior and a discontinuous spatial distribution. They require abundant water, especially *F. angustifolia *which is often found in low elevation riparian forests, particularly in central France [59,60]. Both are protogynous, although anther dehiscence occurs while the stigma is still receptive, and pollen and fruits are wind-dispersed. *Fraxinus excelsior *has a complex trioecious breeding system: sexual types vary across a continuum from pure male individuals to pure females, with all kinds of hermaphrodites in between [47,55,61]. Much less is known regarding the mating system of *F. angustifolia*, which rather seems to be androdioecious [28]. The species differ in their phenology: initiation of flowering occurs between mid-March and early April for *F. excelsior*, and between mid-December and late January for *F. angustifolia*, with larger among-year variation [26,27].

The study site is an unmanaged, naturally regenerated population covering almost 7 ha, located at Saint-Dyé-sur-Loire (latitude 47° 39' 10" N, longitude 1° 28' 40" E, elevation 78 m.a.s.). It is along the Loire river in central France in a zone of sympatry between the two species (see [28] for all details on the stand along with a map). The population was composed of 269 flowering adult trees, and we observed a unimodal distribution of flowering dates, which ranged between the extreme phenologies of the two species. All trees were assigned to one of five phenological groups according to their flowering date (i.e. bud flush, just before pollen offset, 1 group = 2 weeks). These five phenological classes were validated by a Canonical Discriminant Analysis on morphological traits and microsatellite allelic frequencies, and by linkage disequilibrium estimation within and among groups ([28] and Table 4). We also measured the diameter at breast height (DBH), and grouped them into five discrete classes (< 40 cm to > 160 cm, with 40 cm intervals). We finally measured for each individual two fitness components, the flowering and fruiting intensity (each as a visually-assessed 5-level class variable), as well as the intensity of floral gall attacks (also as a visually-assessed 5-level class variable: class 0 includes trees without any gall whereas in class 4, more than 80% of flowers were infected) (see Table 4). For each individual, we also recorded its sexual type, according to one of the four following categories: MM, MH, HM, HH, which correspond either to pure males (MM) or to three types of hermaphrodites, with either a majority of male flowers (MH) or a majority of hermaphroditic flowers (HM) or only hermaphroditic flowers (HH). We followed the classification of Morand-Prieur [30], who also observed three additional categories of trees (three types of females) that we did not observe here.

We harvested 432 seeds on 27 fruiting trees (16 seeds per tree) in the autumn of 2003. The distances between the sampled mother trees ranged from 14 to 1775 m, with a mean of 538 m. We tried to sample individuals of each phenological group, but the earliest flowering trees (first group) did not produce sufficient numbers of fruits. Seeds were rehydrated and sterilized as described in Raquin et al. [62], and embryonic tissues were dehydrated in a 1:1 ethanol-acetone solution. Total DNA was extracted from dried tissue using a DNeasy^® ^96 Plant Kit (Qiagen).

All flowering individuals in the population and all seeds were genotyped at eight microsatellite loci: M-230, FEMSATL 4, 8, 10, 11, 12, 16 and 19 [63,64], under conditions described by Morand et al. [65] and Gérard et al. [28]. The theoretic exclusion probability over the eight loci was 0.9995 for the 269 flowering adult trees.

### Mating model analysis

Paternity analysis allows one to gauge the heterogeneity in male fecundity by estimating the effect of ecological and/or phenotypic variables on this fecundity: for example the effect of the floral phenotype [66], of the inflorescence morphology [67] or of the floral phenology [37]. Here we adapted a mating model developed by Oddou-Muratorio et al. [24], which stems from the neighbourhood model [31,32]. This method allows one to estimate jointly the dispersal curve, level of pollen immigration, selfing rate and the heterogeneity in male fertility. It avoids type I and type II errors occurring in categorical paternity assignment (for a review see [68]).

#### Modeling pollen clouds

The model considers that each offspring *o *sampled from a given mother *j*_*o *_can result either from: (i) self-fertilization (with probability *s*), (ii) cross-pollination by a male located outside the study population (with probability *m*), or (iii) cross-pollination by a male sampled within the study area (with probability 1- *m*- *s*).

The probability that an offspring *o *of mother *j*_*o *_results from self-fertilization and has genotype *g*_*o *_depends on inheritance probabilities only:

*P*_*self *_(*o*, *j*_*o*_) = *sT*(*g*_*o*_|gjo
 MathType@MTEF@5@5@+=feaafiart1ev1aaatCvAUfKttLearuWrP9MDH5MBPbIqV92AaeXatLxBI9gBaebbnrfifHhDYfgasaacH8akY=wiFfYdH8Gipec8Eeeu0xXdbba9frFj0=OqFfea0dXdd9vqai=hGuQ8kuc9pgc9s8qqaq=dirpe0xb9q8qiLsFr0=vr0=vr0dc8meaabaqaciaacaGaaeqabaqabeGadaaakeaacqWGNbWzdaWgaaWcbaGaemOAaO2aaSbaaWqaaiabd+gaVbqabaaaleqaaaaa@312B@, gjo
 MathType@MTEF@5@5@+=feaafiart1ev1aaatCvAUfKttLearuWrP9MDH5MBPbIqV92AaeXatLxBI9gBaebbnrfifHhDYfgasaacH8akY=wiFfYdH8Gipec8Eeeu0xXdbba9frFj0=OqFfea0dXdd9vqai=hGuQ8kuc9pgc9s8qqaq=dirpe0xb9q8qiLsFr0=vr0=vr0dc8meaabaqaciaacaGaaeqabaqabeGadaaakeaacqWGNbWzdaWgaaWcbaGaemOAaO2aaSbaaWqaaiabd+gaVbqabaaaleqaaaaa@312B@)     (1)

where *T*(*g*_*o*_|gjo
 MathType@MTEF@5@5@+=feaafiart1ev1aaatCvAUfKttLearuWrP9MDH5MBPbIqV92AaeXatLxBI9gBaebbnrfifHhDYfgasaacH8akY=wiFfYdH8Gipec8Eeeu0xXdbba9frFj0=OqFfea0dXdd9vqai=hGuQ8kuc9pgc9s8qqaq=dirpe0xb9q8qiLsFr0=vr0=vr0dc8meaabaqaciaacaGaaeqabaqabeGadaaakeaacqWGNbWzdaWgaaWcbaGaemOAaO2aaSbaaWqaaiabd+gaVbqabaaaleqaaaaa@312B@, gjo
 MathType@MTEF@5@5@+=feaafiart1ev1aaatCvAUfKttLearuWrP9MDH5MBPbIqV92AaeXatLxBI9gBaebbnrfifHhDYfgasaacH8akY=wiFfYdH8Gipec8Eeeu0xXdbba9frFj0=OqFfea0dXdd9vqai=hGuQ8kuc9pgc9s8qqaq=dirpe0xb9q8qiLsFr0=vr0=vr0dc8meaabaqaciaacaGaaeqabaqabeGadaaakeaacqWGNbWzdaWgaaWcbaGaemOAaO2aaSbaaWqaaiabd+gaVbqabaaaleqaaaaa@312B@) is the Mendelian segregation probability [69] of the offspring genotype *g*_*o *_given the mother genotype gjo
 MathType@MTEF@5@5@+=feaafiart1ev1aaatCvAUfKttLearuWrP9MDH5MBPbIqV92AaeXatLxBI9gBaebbnrfifHhDYfgasaacH8akY=wiFfYdH8Gipec8Eeeu0xXdbba9frFj0=OqFfea0dXdd9vqai=hGuQ8kuc9pgc9s8qqaq=dirpe0xb9q8qiLsFr0=vr0=vr0dc8meaabaqaciaacaGaaeqabaqabeGadaaakeaacqWGNbWzdaWgaaWcbaGaemOAaO2aaSbaaWqaaiabd+gaVbqabaaaleqaaaaa@312B@.

The probability that an offspring *o *of mother *j*_*o *_results from cross-pollination by a male located outside the study area, and is of genotype *g*_*o*_, depends on the allelic frequencies in the "outside" populations as follows:

*P*_*outside *_(*o*, *j*_*o*_, *AF*) = *mT*(*g*_*o*_|gjo
 MathType@MTEF@5@5@+=feaafiart1ev1aaatCvAUfKttLearuWrP9MDH5MBPbIqV92AaeXatLxBI9gBaebbnrfifHhDYfgasaacH8akY=wiFfYdH8Gipec8Eeeu0xXdbba9frFj0=OqFfea0dXdd9vqai=hGuQ8kuc9pgc9s8qqaq=dirpe0xb9q8qiLsFr0=vr0=vr0dc8meaabaqaciaacaGaaeqabaqabeGadaaakeaacqWGNbWzdaWgaaWcbaGaemOAaO2aaSbaaWqaaiabd+gaVbqabaaaleqaaaaa@312B@, *AF*)     (2)

where *AF *corresponds to the allelic frequencies in the immigrant pollen cloud entering the study population. These external allele frequencies were inferred here from the retrieved paternal gametes of all offspring without compatible male parent within the study population, using the software MLTR 3.0 [70].

The probability that an offspring *o *of mother *j*_*o *_results from cross-pollination by a male *l *with genotype *g*_*l *_sampled within the set Ψ of males occurring in the study population, and has the genotype *g*_*o *_depends on the contribution of male *l *to the pollen cloud as follows:

Pinside(o,jo,Ψ)=(1−m−s)∑l∈Ψπjo,l(θd,F,G)T(go|gjo,gl)     (3)
 MathType@MTEF@5@5@+=feaafiart1ev1aaatCvAUfKttLearuWrP9MDH5MBPbIqV92AaeXatLxBI9gBaebbnrfifHhDYfgasaacH8akY=wiFfYdH8Gipec8Eeeu0xXdbba9frFj0=OqFfea0dXdd9vqai=hGuQ8kuc9pgc9s8qqaq=dirpe0xb9q8qiLsFr0=vr0=vr0dc8meaabaqaciaacaGaaeqabaqabeGadaaakeaacqWGqbaudaWgaaWcbaGaemyAaKMaemOBa4Maem4CamNaemyAaKMaemizaqMaemyzaugabeaakiabcIcaOiabd+gaVjabcYcaSiabdQgaQnaaBaaaleaacqWGVbWBaeqaaOGaeiilaWIaeuiQdKLaeiykaKIaeyypa0JaeiikaGIaeGymaeJaeyOeI0IaemyBa0MaeyOeI0Iaem4CamNaeiykaKYaaabuaeaaiiGacqWFapaCdaWgaaWcbaGaemOAaO2aaSbaaWqaaiabd+gaVbqabaWccqGGSaalcqWGSbaBaeqaaOGaeiikaGIae8hUde3aaSbaaSqaaiabdsgaKbqabaGccqGGSaalcqqGgbGrcqGGSaalcqqGhbWrcqGGPaqkcqWGubavcqGGOaakcqWGNbWzdaWgaaWcbaGaem4Ba8gabeaakiabcYha8jabdEgaNnaaBaaaleaacqWGQbGAdaWgaaadbaGaem4Ba8gabeaaaSqabaGccqGGSaalcqWGNbWzdaWgaaWcbaGaemiBaWgabeaakiabcMcaPaWcbaGaemiBaWMaeyicI4SaeuiQdKfabeqdcqGHris5aOGaaCzcaiaaxMaadaqadaqaaiabiodaZaGaayjkaiaawMcaaaaa@7208@

where π_*jk *_(θ_*d*_, F, G) is the expected proportion of pollen emitted by male *k *in the local pollen cloud that fertilizes female *j*, θ_*d *_is a vector of dispersal parameters, F is a vector of fecundity parameters for all phenotypic classes of males (including parasite attack intensity). In addition to Oddou-Muratorio et al. [24], we included here a vector G containing the probabilities of mating between flowering classes (see below). The proportion π_*jk *_depends (i) on the spatial location of trees (dispersal), via the probability *p*_*jk *_that a pollen grain emitted by male *k *travels to mother *j*, computed from the dispersal kernel given below, (ii) on the phenotypic variables, via the fecundity function Φ_*k *_of male *k *and (iii) on flowering phenology (assortative mating) via the probability γ_*kj *_that a pollen grain is emitted by male *k *when the ovules of mother *j *are receptive, following the equation:

πjk=Φkγkjpjk∑l∈ΨMΦlγljpjl     (4)
 MathType@MTEF@5@5@+=feaafiart1ev1aaatCvAUfKttLearuWrP9MDH5MBPbIqV92AaeXatLxBI9gBaebbnrfifHhDYfgasaacH8akY=wiFfYdH8Gipec8Eeeu0xXdbba9frFj0=OqFfea0dXdd9vqai=hGuQ8kuc9pgc9s8qqaq=dirpe0xb9q8qiLsFr0=vr0=vr0dc8meaabaqaciaacaGaaeqabaqabeGadaaakeaaiiGacqWFapaCdaWgaaWcbaGaemOAaOMaem4AaSgabeaakiabg2da9maalaaabaGaeuOPdy0aaSbaaSqaaiabdUgaRbqabaGccqWFZoWzdaWgaaWcbaGaem4AaSMaemOAaOgabeaakiabdchaWnaaBaaaleaacqWGQbGAcqWGRbWAaeqaaaGcbaWaaabuaeaacqqHMoGrdaWgaaWcbaGaemiBaWgabeaakiab=n7aNnaaBaaaleaacqWGSbaBcqWGQbGAaeqaaOGaemiCaa3aaSbaaSqaaiabdQgaQjabdYgaSbqabaaabaGaemiBaWMaeyicI4SaeuiQdK1aaSbaaWqaaiabd2eanbqabaaaleqaniabggHiLdaaaOGaaCzcaiaaxMaadaqadaqaaiabisda0aGaayjkaiaawMcaaaaa@5624@

The *p*_*jk*_'s, Φ_*k*_'s and γ_*kj*_'s are computed respectively from the dispersal kernel and the F and G vectors (see below).

##### Model for the dispersal kernel

We modeled pollen dispersal using a dispersal kernel *p*(*.;x, y*) that describes the probability for a pollen grain emitted in (0,0) to participate in the pollen cloud fertilizing a tree in (x, y) [7,42]. We used the family of exponential power functions:

p(θd;x,y)=b2πa2Γ(2/b)exp⁡[−(x2+y2a)b]     (5)
 MathType@MTEF@5@5@+=feaafiart1ev1aaatCvAUfKttLearuWrP9MDH5MBPbIqV92AaeXatLxBI9gBaebbnrfifHhDYfgasaacH8akY=wiFfYdH8Gipec8Eeeu0xXdbba9frFj0=OqFfea0dXdd9vqai=hGuQ8kuc9pgc9s8qqaq=dirpe0xb9q8qiLsFr0=vr0=vr0dc8meaabaqaciaacaGaaeqabaqabeGadaaakeaacqWGWbaCcqGGOaakiiGacqWF4oqCdaWgaaWcbaGaemizaqgabeaakiabcUda7iabdIha4jabcYcaSiabdMha5jabcMcaPiabg2da9maalaaabaGaemOyaigabaGaeGOmaiJae8hWdaNaemyyae2aaWbaaSqabeaacqaIYaGmaaGccqqHtoWrcqGGOaakcqaIYaGmcqGGVaWlcqWGIbGycqGGPaqkaaGagiyzauMaeiiEaGNaeiiCaa3aamWaaeaacqGHsisldaqadaqaamaalaaabaWaaOaaaeaacqWG4baEdaahaaWcbeqaaiabikdaYaaakiabgUcaRiabdMha5naaCaaaleqabaGaeGOmaidaaaqabaaakeaacqWGHbqyaaaacaGLOaGaayzkaaWaaWbaaSqabeaacqWGIbGyaaaakiaawUfacaGLDbaacaWLjaGaaCzcamaabmaabaGaeGynaudacaGLOaGaayzkaaaaaa@5B15@

where Γ is the gamma function [71]. θ_*d *_includes the parameters *a *and *b *for an exponential power dispersal kernel, but only *a *for a Gaussian or exponential dispersal kernel (for which *b *is set at 2 or 1 respectively, see [42]). The shape parameter *b *affects the tail of the dispersal function and the scale parameter *a *is homogeneous to a distance [72]. The mean distance δ traveled by a pollen grain under the kernel *p*(*a*, *b*) is given by:

δ=aΓ(3/b)Γ(2/b)     (6)
 MathType@MTEF@5@5@+=feaafiart1ev1aaatCvAUfKttLearuWrP9MDH5MBPbIqV92AaeXatLxBI9gBaebbnrfifHhDYfgasaacH8akY=wiFfYdH8Gipec8Eeeu0xXdbba9frFj0=OqFfea0dXdd9vqai=hGuQ8kuc9pgc9s8qqaq=dirpe0xb9q8qiLsFr0=vr0=vr0dc8meaabaqaciaacaGaaeqabaqabeGadaaakeaaiiGacqWF0oazcqGH9aqpcqWGHbqydaWcaaqaaiabfo5ahjabcIcaOiabiodaZiabc+caViabdkgaIjabcMcaPaqaaiabfo5ahjabcIcaOiabikdaYiabc+caViabdkgaIjabcMcaPaaacaWLjaGaaCzcamaabmaabaGaeGOnaydacaGLOaGaayzkaaaaaa@4100@

For *b *< 1, the dispersal kernel is fat-tailed [72], i.e. it decreases more slowly at long distance than an exponential kernel, implying the possibility of long-distance dispersal. For *b *> 1 (e.g. *b *= 2 for the Gaussian model), the dispersal is thin-tailed, which implies much less long-distance dispersal events. For 1 <*b *< 2, the dispersal kernel is leptokurtic (i.e. a distribution with a high peak and fewer long-distance events than the Gaussian one) and it is platykurtic for *b *> 2 (i.e. a flat-topped distribution) (see [42]).

For each dispersal kernel, i.e. for each set of dispersal parameters θ_*d *_= (*a*, *b*), the probability *p*_*jk *_used in equation (4) that a pollen grain emitted by male *k *travels to mother *j *is given by *p*_*jk *_= *p*(θ_*d*_; *x*_*k*_-*x*_*j*_, *y*_*k*_-*y*_*j*_). We thus assume that pollen emitted by each sampled male tree disperses according to the same dispersal kernel *p*(θ_*d*_; *x*, *y*).

##### Model for male fecundities

Male fecundity was modeled following Oddou-Muratorio et al. [24], considering discrete classes for both phenotypic traits and gall attacks. Denote fci
 MathType@MTEF@5@5@+=feaafiart1ev1aaatCvAUfKttLearuWrP9MDH5MBPbIqV92AaeXatLxBI9gBaebbnrfifHhDYfgasaacH8akY=wiFfYdH8Gipec8Eeeu0xXdbba9frFj0=OqFfea0dXdd9vqai=hGuQ8kuc9pgc9s8qqaq=dirpe0xb9q8qiLsFr0=vr0=vr0dc8meaabaqaciaacaGaaeqabaqabeGadaaakeaacqWGMbGzdaqhaaWcbaGaem4yamgabaGaemyAaKgaaaaa@30D8@ is the relative fecundity of a male belonging to the *c*^th ^class of a given trait *i*. The fecundity Φ_*k *_of a male *k *is thus given by fc(k)i
 MathType@MTEF@5@5@+=feaafiart1ev1aaatCvAUfKttLearuWrP9MDH5MBPbIqV92AaeXatLxBI9gBaebbnrfifHhDYfgasaacH8akY=wiFfYdH8Gipec8Eeeu0xXdbba9frFj0=OqFfea0dXdd9vqai=hGuQ8kuc9pgc9s8qqaq=dirpe0xb9q8qiLsFr0=vr0=vr0dc8meaabaqaciaacaGaaeqabaqabeGadaaakeaacqWGMbGzdaqhaaWcbaGaem4yamMaeiikaGIaem4AaSMaeiykaKcabaGaemyAaKgaaaaa@33E9@ where *c*(*k*) is the phenotypic class to which male *k *belongs. We did not consider any interaction effect between phenotypic traits and gall attacks: for example, the fecundity of a male belonging to class *c*(*k*) of flowering intensity (trait 1) and suffering a level *e*(*k*) of gall attacks (trait 5) is thus simply: Φk=fc(k)1∗fe(k)5
 MathType@MTEF@5@5@+=feaafiart1ev1aaatCvAUfKttLearuWrP9MDH5MBPbIqV92AaeXatLxBI9gBaebbnrfifHhDYfgasaacH8akY=wiFfYdH8Gipec8Eeeu0xXdbba9frFj0=OqFfea0dXdd9vqai=hGuQ8kuc9pgc9s8qqaq=dirpe0xb9q8qiLsFr0=vr0=vr0dc8meaabaqaciaacaGaaeqabaqabeGadaaakeaacqqHMoGrdaWgaaWcbaGaem4AaSgabeaakiabg2da9iabdAgaMnaaDaaaleaacqWGJbWycqGGOaakcqWGRbWAcqGGPaqkaeaacqaIXaqmaaGccqGHxiIkcqWGMbGzdaqhaaWcbaGaemyzauMaeiikaGIaem4AaSMaeiykaKcabaGaeGynaudaaaaa@3F6A@. All relative fecundities fci
 MathType@MTEF@5@5@+=feaafiart1ev1aaatCvAUfKttLearuWrP9MDH5MBPbIqV92AaeXatLxBI9gBaebbnrfifHhDYfgasaacH8akY=wiFfYdH8Gipec8Eeeu0xXdbba9frFj0=OqFfea0dXdd9vqai=hGuQ8kuc9pgc9s8qqaq=dirpe0xb9q8qiLsFr0=vr0=vr0dc8meaabaqaciaacaGaaeqabaqabeGadaaakeaacqWGMbGzdaqhaaWcbaGaem4yamgabaGaemyAaKgaaaaa@30D8@'s of the different phenotypic classes of males are stored in the vector F.

##### Models for flowering phenology

We modeled the flowering phenology in two different ways. In *model 1*, we modeled the relative flowering phenology considering the phenological differences between pollen-emitting and pollen-recipient trees to assess the strength of assortative mating. In that case, we define *g*_*d *_as the relative male success of a tree at siring offspring on another tree when the phenological difference between the two trees is *d*. The relative male fecundity γ_*kj *_of tree *k *on tree *j *is thus *g*_*d*(*kj*)_, where *d*(*kj*) is the difference in phenology between pollen-emitting tree *k *and pollen-recipient tree *j*, *d*(*kj*) being zero if *k *and *j *flower simultaneously, positive if the pollen-emitting tree *k *flowers before the pollen-recipient tree *j *and negative if *k *flowers after *j*. The vector G contains all *g*_*d *_'s.

In *model 2*, we applied to the phenology the approach described above for other phenotypic traits to assess the overall relative male mating success of the different phenological groups on all mothers of the population. In that case, the vector G, containing the relative fecundities of the different phenological groups, is defined in the same manner as F.

#### Joint estimation of the dispersal kernel, male fecundities and phenological assortative mating

We used a maximum likelihood approach to estimate jointly the vector of dispersal parameters (*θ*_*d*_), the vector of the relative male fecundities of the different phenotypic classes and infection intensities (F), the vector of the relative fecundities of different classes of flowering time or flowering time differences (G), the selfing rate (*s*) and pollen immigration rate (*m*). The log-likelihood function of all observed progenies collected from females, given the above model is given by:

log⁡L(θd,F,G,s,m)=∑o=1olog⁡[Pself(o,jo)+Pinside(o,jo,Ψ)+Poutside(o,jo,AF)]     (7)
 MathType@MTEF@5@5@+=feaafiart1ev1aaatCvAUfKttLearuWrP9MDH5MBPbIqV92AaeXatLxBI9gBaebbnrfifHhDYfgasaacH8akY=wiFfYdH8Gipec8Eeeu0xXdbba9frFj0=OqFfea0dXdd9vqai=hGuQ8kuc9pgc9s8qqaq=dirpe0xb9q8qiLsFr0=vr0=vr0dc8meaabaqaciaacaGaaeqabaqabeGadaaakeaacyGGSbaBcqGGVbWBcqGGNbWzcqWGmbatcqGGOaakiiGacqWF4oqCdaWgaaWcbaGaemizaqgabeaakiabcYcaSiabbAeagjabcYcaSiabbEeahjabcYcaSiabdohaZjabcYcaSiabd2gaTjabcMcaPiabg2da9maaqahabaGagiiBaWMaei4Ba8Maei4zaCgaleaacqWGVbWBcqGH9aqpcqaIXaqmaeaacqWGVbWBa0GaeyyeIuoakmaadmaabaGaemiuaa1aaSbaaSqaaiabdohaZjabdwgaLjabdYgaSjabdAgaMbqabaGccqGGOaakcqWGVbWBcqGGSaalcqWGQbGAdaWgaaWcbaGaem4Ba8gabeaakiabcMcaPiabgUcaRiabdcfaqnaaBaaaleaacqWGPbqAcqWGUbGBcqWGZbWCcqWGPbqAcqWGKbazcqWGLbqzaeqaaOGaeiikaGIaem4Ba8MaeiilaWIaemOAaO2aaSbaaSqaaiabd+gaVbqabaGccqGGSaalcqqHOoqwcqGGPaqkcqGHRaWkcqWGqbaudaWgaaWcbaGaem4Ba8MaemyDauNaemiDaqNaem4CamNaemyAaKMaemizaqMaemyzaugabeaakiabcIcaOiabd+gaVjabcYcaSiabdQgaQnaaBaaaleaacqWGVbWBaeqaaOGaeiilaWIaemyqaeKaemOrayKaeiykaKcacaGLBbGaayzxaaGaaCzcaiaaxMaadaqadaqaaiabiEda3aGaayjkaiaawMcaaaaa@88D2@

Equation (7) assumes that all fertilization events are independent of each other.

#### Statistical analyses

All analyses described below were performed using Mathematica 5.0 (Wolfram Research Inc.). Notebooks are available upon request from PRG.

##### Fits

All fits were achieved by maximizing the log-likelihood of equation (7) following a quasi-Newton algorithm. For the exponential power dispersal kernel parameters, we estimated the mean dispersal distance δ and the shape parameter *b *rather than *a *and *b*. To ensure numerical convergence, parameters *m *and *s *were transformed through a logit function [*m *= e^*m*'^/(1+e^*m*'^) and *s *= e^*s*'^/(1+e^*s*'^)] and male fecundity parameters were transformed through fc(k)=10f′c(k)
 MathType@MTEF@5@5@+=feaafiart1ev1aaatCvAUfKttLearuWrP9MDH5MBPbIqV92AaeXatLxBI9gBaebbnrfifHhDYfgasaacH8akY=wiFfYdH8Gipec8Eeeu0xXdbba9frFj0=OqFfea0dXdd9vqai=hGuQ8kuc9pgc9s8qqaq=dirpe0xb9q8qiLsFr0=vr0=vr0dc8meaabaqaciaacaGaaeqabaqabeGadaaakeaacqWGMbGzdaWgaaWcbaGaem4yamMaeiikaGIaem4AaSMaeiykaKcabeaakiabg2da9iabigdaXiabicdaWmaaCaaaleqabaGafmOzayMbauaadaWgaaadbaGaem4yamMaeiikaGIaem4AaSMaeiykaKcabeaaaaaaaa@3B96@.

##### Confidence intervals

We obtained the 95% likelihood-profile confidence intervals for δ and *b*, by plotting contour plots of the likelihood function [73]. For the vectors of parameters F and G (male fecundities and flowering phenology), and for the parameters *m *and *s*, we derived 95% confidence intervals by computing the asymptotic Gaussian variance-covariance matrix, which is the inverse of the Fisher's information matrix (i.e. the opposite of the expectation of second-order partial derivatives of the log-likelihood function with respect to all couples of parameters) [73]. As these parameters were estimated through a transformation function, we first computed a symmetric Gaussian asymptotic interval for the transformed parameters (*m*', *s*' and *f*') by using a delta method [73]. We then obtained asymmetric confidence intervals for the parameters of interest (*m*, *s*, and *f*) by the reverse transformation of the bounds of the intervals.

##### Tests

We tested for the effect of dispersal kernel, spatial and temporal non-random mating, and of the variation in phenotypic traits and gall attacks on male mating success, by building several corresponding nested models from equation (4) and using likelihood-ratio tests (LRT) in a type III approach [24].

#### Effect of genotyping error

To account for possible genotyping error, we re-computed the Mendelian transition probabilities allowing an error of ± 1 microsatellite repeat unit for genotypes of all males at all loci (see Appendix). All models described above were then used with the modified log-likelihood accounting for a fixed mistyping rate, either a low rate of 0.01%, or a relatively high rate of 2.5%.

### Effective male population density

The reduction in effective male population density can have strong consequences: it may for example increase drift in natural populations and influence the rate of accumulation of beneficial and deleterious mutations [24,74]. In fact, unequal male fecundities and asynchronous flowering may drastically reduce the effective male population density [74]. Using male fecundities estimated as described above, we thus estimated the reduction in effective male population density caused by heterogeneity in male fecundity under the complete model following Oddou-Muratorio et al. [24]. This density is defined by *d*_*em *_= *N*_*em *_/*A*, where *N*_*em *_is the effective male population size (i.e. the inverse of the probability that two pollen grains come from the same male) and *A *is the area covered by the study population. The reduction in effective male population density can be expressed as:

demdobs=1N(∑k∈Ψ(∏i=1nfc(k)i))2∑k∈Ψ(∏i=1nfc(k)i)2     (8)
 MathType@MTEF@5@5@+=feaafiart1ev1aaatCvAUfKttLearuWrP9MDH5MBPbIqV92AaeXatLxBI9gBaebbnrfifHhDYfgasaacH8akY=wiFfYdH8Gipec8Eeeu0xXdbba9frFj0=OqFfea0dXdd9vqai=hGuQ8kuc9pgc9s8qqaq=dirpe0xb9q8qiLsFr0=vr0=vr0dc8meaabaqaciaacaGaaeqabaqabeGadaaakeaadaWcaaqaaiabdsgaKnaaBaaaleaacqWGLbqzcqWGTbqBaeqaaaGcbaGaemizaq2aaSbaaSqaaiabd+gaVjabdkgaIjabdohaZbqabaaaaOGaeyypa0ZaaSaaaeaacqaIXaqmaeaacqWGobGtaaWaaSaaaeaadaqadaqaamaaqafabaWaaeWaaeaadaqeWbqaaiabdAgaMnaaDaaaleaacqWGJbWycqGGOaakcqWGRbWAcqGGPaqkaeaacqWGPbqAaaaabaGaemyAaKMaeyypa0JaeGymaedabaGaemOBa4ganiabg+GivdaakiaawIcacaGLPaaaaSqaaiabdUgaRjabgIGiolabfI6azbqab0GaeyyeIuoaaOGaayjkaiaawMcaamaaCaaaleqabaGaeGOmaidaaaGcbaWaaabuaeaadaqadaqaamaarahabaGaemOzay2aa0baaSqaaiabdogaJjabcIcaOiabdUgaRjabcMcaPaqaaiabdMgaPbaaaeaacqWGPbqAcqGH9aqpcqaIXaqmaeaacqWGUbGBa0Gaey4dIunaaOGaayjkaiaawMcaamaaCaaaleqabaGaeGOmaidaaaqaaiabdUgaRjabgIGiolabfI6azbqab0GaeyyeIuoaaaGccaWLjaGaaCzcamaabmaabaGaeGioaGdacaGLOaGaayzkaaaaaa@6E05@

where *d*_*obs *_is the observed male population density, fc(k)i
 MathType@MTEF@5@5@+=feaafiart1ev1aaatCvAUfKttLearuWrP9MDH5MBPbIqV92AaeXatLxBI9gBaebbnrfifHhDYfgasaacH8akY=wiFfYdH8Gipec8Eeeu0xXdbba9frFj0=OqFfea0dXdd9vqai=hGuQ8kuc9pgc9s8qqaq=dirpe0xb9q8qiLsFr0=vr0=vr0dc8meaabaqaciaacaGaaeqabaqabeGadaaakeaacqWGMbGzdaqhaaWcbaGaem4yamMaeiikaGIaem4AaSMaeiykaKcabaGaemyAaKgaaaaa@33E9@ is the fecundity of male *k *belonging to the *c*^th ^class of the phenotypic trait *i*, and *n *is the number of traits under consideration. We also estimated the reduction in effective male population density due to dispersal features and phenology, including in the above sums all *p*_*jk*_'s and all *g*_*d*(*kj*)_'s.

### Estimation of selfing rates within phenological groups

We performed a mixed-mating model analysis using the software MLTR 3.0 [70], to assess the relation between selfing rate and flowering time, by estimating the mean outcrossing rates *t*_*m *_in each phenological group (from the second to the fifth, because we had no seeds for the first group) from the multilocus genotypes of the seeds harvested on the mother trees, using an EM algorithm. Standard errors were computed by performing 1000 bootstrap replicates using families as resampling units. We also estimated the family-level *t*_*m *_values (i.e. the mean outcrossing rates among seeds harvested from each mother-tree) [70].

## Authors' contributions

NFL initiated the project. PRG and JFFM conceived the sampling design, collected samples and field data. PRG carried out the microsatellite genotyping. PRG, EKK and FA developed the statistical model adapted to this data set. PRG, FA, EKK, JFFM and NFL interpreted the results and wrote the paper. All authors read and approved the final manuscript.

## Appendix

### Modelling the genotyping error rate

If α was the error rate and if the paternal allele of the offspring was unambiguously known, we assigned as father with a probability α, a male homozygous for an allele that differ by 1 repeat unit from the offspring, and with a probability 1–2α, a male homozygous for the same allele.

This means that in equation 8, the standard transition probabilities *T*(*g*_*o*_|gjo
 MathType@MTEF@5@5@+=feaafiart1ev1aaatCvAUfKttLearuWrP9MDH5MBPbIqV92AaeXatLxBI9gBaebbnrfifHhDYfgasaacH8akY=wiFfYdH8Gipec8Eeeu0xXdbba9frFj0=OqFfea0dXdd9vqai=hGuQ8kuc9pgc9s8qqaq=dirpe0xb9q8qiLsFr0=vr0=vr0dc8meaabaqaciaacaGaaeqabaqabeGadaaakeaacqWGNbWzdaWgaaWcbaGaemOAaO2aaSbaaWqaaiabd+gaVbqabaaaleqaaaaa@312B@, *g*_*l*_) defined by Meagher (1986) were replaced by T(go|gjo,gl)=∏lociTloc(go,loc|gjo,loc,gl,loc)
 MathType@MTEF@5@5@+=feaafiart1ev1aaatCvAUfKttLearuWrP9MDH5MBPbIqV92AaeXatLxBI9gBaebbnrfifHhDYfgasaacH8akY=wiFfYdH8Gipec8Eeeu0xXdbba9frFj0=OqFfea0dXdd9vqai=hGuQ8kuc9pgc9s8qqaq=dirpe0xb9q8qiLsFr0=vr0=vr0dc8meaabaqaciaacaGaaeqabaqabeGadaaakeaacqWGubavcqGGOaakcqWGNbWzdaWgaaWcbaGaem4Ba8gabeaakiabcYha8jabdEgaNnaaBaaaleaacqWGQbGAdaWgaaadbaGaem4Ba8gabeaaaSqabaGccqGGSaalcqWGNbWzdaWgaaWcbaGaemiBaWgabeaakiabcMcaPiabg2da9maarafabaGaemivaq1aaSbaaSqaaiabdYgaSjabd+gaVjabdogaJbqabaGccqGGOaakcqWGNbWzdaWgaaWcbaGaem4Ba8MaeiilaWIaemiBaWMaem4Ba8Maem4yamgabeaakiabcYha8jabdEgaNnaaBaaaleaacqWGQbGAdaWgaaadbaGaem4Ba8gabeaaliabcYcaSiabdYgaSjabd+gaVjabdogaJbqabaGccqGGSaalcqWGNbWzdaWgaaWcbaGaemiBaWMaeiilaWIaemiBaWMaem4Ba8Maem4yamgabeaakiabcMcaPaWcbaGaemiBaWMaem4Ba8Maem4yamMaemyAaKgabeqdcqGHpis1aaaa@67C4@, where for each locus *loc*, and with the notations from [70]:

Tloc({o1,o2}|{m1,m2},{f1,f2})=Do1m1m2D˜o2f1f2+Do2m1m2D˜o1f1f2(1+δo1o2),
 MathType@MTEF@5@5@+=feaafiart1ev1aaatCvAUfKttLearuWrP9MDH5MBPbIqV92AaeXatLxBI9gBaebbnrfifHhDYfgasaacH8akY=wiFfYdH8Gipec8Eeeu0xXdbba9frFj0=OqFfea0dXdd9vqai=hGuQ8kuc9pgc9s8qqaq=dirpe0xb9q8qiLsFr0=vr0=vr0dc8meaabaqaciaacaGaaeqabaqabeGadaaakeaacqWGubavdaWgaaWcbaGaemiBaWMaem4Ba8Maem4yamgabeaakmaabmaabaGaei4EaSNaem4Ba82aaSbaaSqaaiabigdaXaqabaGccqGGSaalcqWGVbWBdaWgaaWcbaGaeGOmaidabeaakiabc2ha9jabcYha8jabcUha7jabd2gaTnaaBaaaleaacqaIXaqmaeqaaOGaeiilaWIaemyBa02aaSbaaSqaaiabikdaYaqabaGccqGG9bqFcqGGSaalcqGG7bWEcqWGMbGzdaWgaaWcbaGaeGymaedabeaakiabcYcaSiabdAgaMnaaBaaaleaacqaIYaGmaeqaaOGaeiyFa0hacaGLOaGaayzkaaGaeyypa0ZaaSaaaeaacqWGebardaqhaaWcbaGaem4Ba82aaSbaaWqaaiabigdaXaqabaaaleaacqWGTbqBdaWgaaadbaGaeGymaedabeaaliabd2gaTnaaBaaameaacqaIYaGmaeqaaaaakiqbdseaezaaiaWaa0baaSqaaiabd+gaVnaaBaaameaacqaIYaGmaeqaaaWcbaGaemOzay2aaSbaaWqaaiabigdaXaqabaWccqWGMbGzdaWgaaadbaGaeGOmaidabeaaaaGccqGHRaWkcqWGebardaqhaaWcbaGaem4Ba82aaSbaaWqaaiabikdaYaqabaaaleaacqWGTbqBdaWgaaadbaGaeGymaedabeaaliabd2gaTnaaBaaameaacqaIYaGmaeqaaaaakiqbdseaezaaiaWaa0baaSqaaiabd+gaVnaaBaaameaacqaIXaqmaeqaaaWcbaGaemOzay2aaSbaaWqaaiabigdaXaqabaWccqWGMbGzdaWgaaadbaGaeGOmaidabeaaaaaakeaadaqadaqaaiabigdaXiabgUcaRGGaciab=r7aKnaaBaaaleaacqWGVbWBdaWgaaadbaGaeGymaedabeaaliabd+gaVnaaBaaameaacqaIYaGmaeqaaaWcbeaaaOGaayjkaiaawMcaaaaacqGGSaalaaa@818A@

with δ_*ij *_= 1 if i = j and 0 if i ≠ j, Dom1m2=(δom1+δom22)
 MathType@MTEF@5@5@+=feaafiart1ev1aaatCvAUfKttLearuWrP9MDH5MBPbIqV92AaeXatLxBI9gBaebbnrfifHhDYfgasaacH8akY=wiFfYdH8Gipec8Eeeu0xXdbba9frFj0=OqFfea0dXdd9vqai=hGuQ8kuc9pgc9s8qqaq=dirpe0xb9q8qiLsFr0=vr0=vr0dc8meaabaqaciaacaGaaeqabaqabeGadaaakeaacqWGebardaqhaaWcbaGaem4Ba8gabaGaemyBa02aaSbaaWqaaiabigdaXaqabaWccqWGTbqBdaWgaaadbaGaeGOmaidabeaaaaGccqGH9aqpdaqadaqaamaalaaabaacciGae8hTdq2aaSbaaSqaaiabd+gaVjabd2gaTnaaBaaameaacqaIXaqmaeqaaaWcbeaakiabgUcaRiab=r7aKnaaBaaaleaacqWGVbWBcqWGTbqBdaWgaaadbaGaeGOmaidabeaaaSqabaaakeaacqaIYaGmaaaacaGLOaGaayzkaaaaaa@4479@ is the Mendelian contribution from the mother, without any mistyping, and the contribution from the father accounting for possible mistyping:

D˜of1f2=((αδof1+1+αδof1−1+(1−2α)δof1)+(αδof2+1+αδof2−1+(1−2α)δof2)2)
 MathType@MTEF@5@5@+=feaafiart1ev1aaatCvAUfKttLearuWrP9MDH5MBPbIqV92AaeXatLxBI9gBaebbnrfifHhDYfgasaacH8akY=wiFfYdH8Gipec8Eeeu0xXdbba9frFj0=OqFfea0dXdd9vqai=hGuQ8kuc9pgc9s8qqaq=dirpe0xb9q8qiLsFr0=vr0=vr0dc8meaabaqaciaacaGaaeqabaqabeGadaaakeaacuWGebargaacamaaDaaaleaacqWGVbWBaeaacqWGMbGzdaWgaaadbaGaeGymaedabeaaliabdAgaMnaaBaaameaacqaIYaGmaeqaaaaakiabg2da9maabmaabaWaaSaaaeaacqGGOaakiiGacqWFXoqycqWF0oazdaWgaaWcbaGaem4Ba8MaemOzay2aaSbaaWqaaiabigdaXaqabaWccqGHRaWkcqaIXaqmaeqaaOGaey4kaSIae8xSdeMae8hTdq2aaSbaaSqaaiabd+gaVjabdAgaMnaaBaaameaacqaIXaqmaeqaaSGaeyOeI0IaeGymaedabeaakiabgUcaRiabcIcaOiabigdaXiabgkHiTiabikdaYiab=f7aHjabcMcaPiab=r7aKnaaBaaaleaacqWGVbWBcqWGMbGzdaWgaaadbaGaeGymaedabeaaaSqabaGccqGGPaqkcqGHRaWkcqGGOaakcqWFXoqycqWF0oazdaWgaaWcbaGaem4Ba8MaemOzay2aaSbaaWqaaiabikdaYaqabaWccqGHRaWkcqaIXaqmaeqaaOGaey4kaSIae8xSdeMae8hTdq2aaSbaaSqaaiabd+gaVjabdAgaMnaaBaaameaacqaIYaGmaeqaaSGaeyOeI0IaeGymaedabeaakiabgUcaRiabcIcaOiabigdaXiabgkHiTiabikdaYiab=f7aHjabcMcaPiab=r7aKnaaBaaaleaacqWGVbWBcqWGMbGzdaWgaaadbaGaeGOmaidabeaaaSqabaGccqGGPaqkaeaacqaIYaGmaaaacaGLOaGaayzkaaaaaa@7C24@

## References

[B1] Arnold ML, May RM, Harvey PH (1997). Natural hybridization and evolution. Oxford Series in Ecology and Evolution.

[B2] Barton NH (2001). The role of hybridization in evolution. Mol Ecol.

[B3] Coyne JA, Orr HA (2004). Speciation.

[B4] Rundle HD, Nosil P (2005). Ecological speciation. Ecol Letters.

[B5] Austerlitz F, Mariette S, Machon N, Gouyon PH, Godelle B (2000). Effects of colonization processes on genetic diversity: differences between annual plants and tree species. Genetics.

[B6] Austerlitz F, Garnier-Géré P (2003). Modelling the impact of colonization on genetic diversity and differentiation of forest trees: interaction of life cycle, pollen flow and seed long-distance dispersal. Heredity.

[B7] Klein EK, Lavigne C, Gouyon PH (2006). Mixing of propagules from discrete sources at long distance: comparing a dispersal tail to an exponential. BMC Ecol.

[B8] Sork VL, Nason J, Campbell DR, Fernandez JF (1999). Landscape approaches to historical and contemporary gene flow in plants. Trends Ecol Evol.

[B9] Cheptou PO, Lepart J, Escarre J (2002). Mating system variation along a successional gradient in the allogamous and colonizing plant Crepis sancta (Asteraceae). J Evol Biol.

[B10] Goodwillie C, Kalisz S, Eckert CG (2005). The evolutionary enigma of mixed mating systems in plants: occurrence, theoretical explanations, and empirical evidence. Annu Rev Ecol Evol Syst.

[B11] Jarne P, Charlesworth D (1993). The evolution of the selfing rate in functionally hermaphrodite plants and animals. Annu Rev Ecol Syst.

[B12] McNeilly T, Antonovics J (1968). Evolution in closely adjacent plant populations. IV. Barriers to gene flow. Heredity.

[B13] Silvertown J, Servaes C, Biss P, Macleod D (2005). Reinforcement of reproductive isolation between adjacent populations in the Park Grass Experiment. Heredity.

[B14] Lamont BB, He T, Enright NJ, Krauss SL, Miller BP (2003). Anthropogenic disturbance promotes hybridization between Banksia species by altering their biology. J Evol Biol.

[B15] Hendry AP, Day T (2005). Population structure attributable to reproductive time: isolation by time and adaptation by time. Mol Ecol.

[B16] Fox GA (2003). Assortative mating and plant phenology: evolutionary and practical consequences. Evol Ecol Res.

[B17] Weis AE, Winterer J, Vacher C, Kossler TM, Young CA, Le Buhn GL (2005). Phenological assortative mating in flowering plants: the nature and consequences of its frequency-dependence. Evol Ecol Res.

[B18] Cruzan MB, Arnold ML (1994). Assortative mating and natural selection in an Iris hybrid zone. Evolution.

[B19] Bacilieri R, Ducousso A, Petit RJ, Kremer A (1996). Mating system and asymmetric hybridization in a mixed stand of european oaks. Evolution.

[B20] Cornman RS, Burke JM, Wesselingh RA, Arnold ML (2004). Contrasting genetic structure of adults and progeny in a Louisiana Iris hybrid population. Evolution.

[B21] Valbuena-Carabaña M, González-Martínez SC, Sork VL, Collada C, Soto A, Goicoechea PG, Gil L (2005). Gene flow and hybridization in a mixed oak forest (Quercus pyrenaica Willd. and Quercus petraea (Matts.) Liebl.) in central Spain. Heredity.

[B22] Nürnberger BD, Barton NH, Kruuk LEB, Vines TH (2005). Mating patterns in a hybrid zone of fire-bellied toads (Bombina): inferences from adult and full-sib genotypes. Heredity.

[B23] Weis AE (2005). Direct and indirect assortative mating: a multivariate approach to plant flowering schedules. J Evol Biol.

[B24] Oddou-Muratorio S, Klein EK, Austerlitz F (2005). Pollen flow in the wildservice tree, Sorbus torminalis (L.) Crantz. II. Pollen dispersal and heterogeneity in mating success inferred from parent–offspring analysis. Mol Ecol.

[B25] Fernandez-Manjarrés JF, Gérard PR, Dufour J, Raquin C, Frascaria-Lacoste N (2006). Differential patterns of morphological and molecular hybridization between Fraxinus excelsior L. and F. angustifolia Vahl. (Oleaceae) in Eastern and Western France. Mol Ecol.

[B26] Jato V, Rodriguez-Rajo FJ, Dacosta N, Aira MJ (2004). Heat and chill requirements of Fraxinus flowering in Galicia (NW Spain). Grana.

[B27] Raquin C, Brachet S, Jeandroz S, Vedel F, Frascaria-Lacoste N (2002). Combined analyses of microsatellite and RAPD markers demonstrate possible hybridisation between Fraxinus excelsior L. and Fraxinus angustifolia Vahl. For Genet.

[B28] Gérard PR, Fernandez-Manjarrés JF, Frascaria-Lacoste N (2006). Temporal cline in a hybrid zone population between Fraxinus excelsior L. and F. angustifolia Vahl. Mol Ecol.

[B29] Bacles CFE, Burczyk J, Lowe AJ, Ennos RA (2005). Historical and contemporary mating patterns in remnant populations of the forest tree Fraxinus excelsior L.. Evolution.

[B30] Morand-Prieur ME (2003). Evolution et maintien d'un système de reproduction polymorphe. Approche génétique et écologique de la polygamie chez le frêne commun, Fraxinus excelsior L..

[B31] Adams WT, Birkes DS, Fineschi S, Malvolti ME, Cannata F, Hattemer HH (1991). Estimating mating patterns in forest tree populations. Biochemical markers in the population genetics of forest trees.

[B32] Burczyk J, Koralewski TE (2005). Parentage versus two-generation analyses for estimating pollen-mediated gene flow in plant populations. Mol Ecol.

[B33] Araki H, Blouin MS (2005). Unbiased estimation of relative reproductive success of different groups: evaluation and correction of bias caused by parentage assignment errors. Mol Ecol.

[B34] Slavov GT, Howe GT, Gyaourova AV, Birkes DS, Adams WT (2005). Estimating pollen flow using SSR markers and paternity exclusion: accounting for mistyping. Mol Ecol.

[B35] Oddou-Muratorio S, Houot ML, Demesure-Musch B, Austerlitz F (2003). Pollen flow in the wildservice tree, Sorbus torminalis (L.) Crantz. I. Evaluating the paternity analysis procedure in continuous populations. Mol Ecol.

[B36] Marshall TC, Slate J, Kruuk LEB, Pemberton JM (1998). Statistical confidence for likelihood-based paternity inference in natural populations. Mol Ecol.

[B37] Burczyk J, Prat D (1997). Male reproductive success in Pseudotsuga menziesii (Mirb.) Franco: the effects of spatial structure and flowering characteristics. Heredity.

[B38] Streiff R, Ducousso A, Lexer C, Steinkellner H, Gloessl J, Kremer A (1999). Pollen dispersal inferred from paternity analysis in a mixed oak stand of Quercus robur L. and Q. petraea (Matt.) Liebl.. Mol Ecol.

[B39] Nurminiemi M, Tufto J, Nilsson NO, Rognli OA (1998). Spatial models of pollen dispersal in the forage grass meadow fescue. Evol Ecol.

[B40] Hardy OJ, González-Martínez SC, Fréville H, Boquien G, Mignot A, Colas B, Olivieri I (2004). Fine-scale genetic structure and gene dispersal in Centaurea corymbosa (Asteraceae). I. Pattern of pollen dispersal. J Evol Biol.

[B41] Devaux C, Lavigne C, Falentin-Guyomarc'h H, Vautrin S, Lecomte J, Klein EK (2005). High diversity of oilseed rape pollen clouds over an agro-ecosystem indicates long-distance dispersal. Mol Ecol.

[B42] Austerlitz F, Dick CW, Dutech C, Klein EK, Oddou-Muratorio S, Smouse PE, Sork VL (2004). Using genetic markers to estimate the pollen dispersal curve. Mol Ecol.

[B43] Robledo-Arnuncio JJ, Gil L (2005). Patterns of pollen dispersal in a small population of Pinus sylvestris L. revealed by total-exclusion paternity analysis. Heredity.

[B44] Broyles SB (2002). Hybrid bridges to gene flow: a case study in milkweeds (Asclepias). Evolution.

[B45] Porcher E, Lande R (2005). The evolution of self-fertilization and inbreeding depression under pollen discounting and pollen limitation. J Evol Biol.

[B46] Klinkhamer PGL, de Jong TJ, Metz H (1997). Sex and size in cosexual plants. Trends Ecol Evol.

[B47] Morand-Prieur ME, Raquin C, Shykoff JA, Frascaria-Lacoste N (2003). Males outcompete hermaphrodites for seed siring success in controlled crosses in the polygamous Fraxinus excelsior (Oleaceae). Am J Bot.

[B48] Charnov EL (1982). The theory of sex allocation.

[B49] Klinkhamer PGL, De Jong TJ (2005). Evolutionary Ecology of Plant Reproductive Strategies.

[B50] Cadet C, Metz JAJ, Klinkhamer PGL (2004). Size and the not-so-single sex: disentangling the effects of size and budget on sex allocation in hermaphrodites. Am Nat.

[B51] Campbell DR (2000). Experimental tests of sex-allocation theory in plants. Trends Ecol Evol.

[B52] Broyles SB, Wyatt R (1990). Paternity analysis in a natural population of Asclepias exaltata: multiple paternity, functional gender, and the "pollen-donation hypothesis". Evolution.

[B53] Conner JK, Rush S, Kercher S, Jennetten P (1996). Measurements of natural selection on floral traits in wild radish (Raphanus raphanistrum). II. Selection through lifetime male and total fitness. Evolution.

[B54] Devlin B, Ellstrand NC (1990). Male and female fertility variation in wild radish, a hermaphrodite. Am Nat.

[B55] Wardle P (1961). Biological flora of the British Isles. Fraxinus excelsior L.. J Ecol.

[B56] Gérard PR, Fernandez-Manjarrés JF, Bertolino P, Dufour J, Raquin C, Frascaria-Lacoste N (2006). New insights in the recognition of the European ash species Fraxinus excelsior L. and Fraxinus angustifolia Vahl as useful tools for forest management. Ann For Sci.

[B57] Ferdy JB, Austerlitz F (2002). Extinction and introgression in a community of partially cross-fertile plant species. Am Nat.

[B58] Jeandroz S, Roy A, Bousquet J (1997). Phylogeny and phylogeography of the circumpolar genus Fraxinus (Oleaceae) based on Internal Transcribed Spacer sequences of nuclear ribosomal DNA. Mol Phylogenet Evol.

[B59] Picard JF (1983). A propos du frêne oxyphylle, Fraxinus angustifolia Vahl.. Forêt-Entreprise.

[B60] Marigo G, Peltier JP, Girel J, Pautou G (2000). Success in the demographic expansion of Fraxinus excelsior L.. Trees.

[B61] Binggeli P, Power J Gender variation in ash (Fraxinus excelsior L.). http://members.tripod.co.uk/WoodyPlantEcology/species/ash.htm.

[B62] Raquin C, Jung-Muller B, Dufour J, Frascaria-Lacoste N (2002). Rapid seedling obtaining from European ash species Fraxinus excelsior (L.) and Fraxinus angustifolia (Vahl.). Ann For Sci.

[B63] Brachet S, Jubier MF, Richard M, Jung-Muller B, Frascaria-Lacoste N (1999). Rapid identification of microsatellite loci using 5' anchored PCR in the common ash Fraxinus excelsior. Mol Ecol.

[B64] Lefort F, Brachet S, Frascaria-Lacoste N, Edwards KJ, Douglas GC (1999). Identification and characterization of microsatellite loci in ash (Fraxinus excelsior L.) and their conservation in the olive family (Oleaceae). Mol Ecol.

[B65] Morand ME, Brachet S, Rossignol P, Dufour J, Frascaria-Lacoste N (2002). A generalized heterozygote deficiency assessed with microsatellites in French common ash populations. Mol Ecol.

[B66] Elle E, Meagher TR (2000). Sex allocation and reproductive success in the andromonoecious perennial Solanum carolinense. II. Paternity and functional gender. Am Nat.

[B67] Smouse PE, Meagher TR, Kobak CJ (1999). Parentage analysis in Chamaelirium luteum (L.) Gray (Liliaceae): why do some males have higher reproductive contributions?. J Evol Biol.

[B68] Jones AG, Ardren WR (2003). Methods of parentage analysis in natural populations. Mol Ecol.

[B69] Meagher TR (1986). Analysis of paternity within a natural population of Chamaelirium luteum. I. Identification of most-likely male parents. Am Nat.

[B70] Ritland K (2002). Extensions of models for the estimation of mating systems using n independant loci. Heredity.

[B71] Abramowitz M, Stegun IA (1964). Handbook of Mathematical Functions with Formulas, Graphs, and Mathematical Tables.

[B72] Clark JS (1998). Why trees migrate so fast: confronting theory with dispersal biology and the paleorecord. Am Nat.

[B73] Coles S (2001). An Introduction to Statistical Modeling of Extreme Values.

[B74] Robledo-Arnuncio JJ, Austerlitz F, Smouse PE (2006). A new method of estimating the pollen dispersal curve independently of the effective density. Genetics.

